# LTBP1 plays a potential bridge between depressive disorder and glioblastoma

**DOI:** 10.1186/s12967-020-02509-3

**Published:** 2020-10-15

**Authors:** Xiaojun Fu, Pei Zhang, Hongwang Song, Chenxing Wu, Shengzhen Li, Shouwei Li, Changxiang Yan

**Affiliations:** 1 Department of Neurosurgery, Sanbo Brain Hospital, Capital Medical University, Xiangshanyikesong 50#, HaiDian District, Beijing, 100093 China; 2 Capital Medical University, Beijing, People’s Republic of China; 3 Beijing Institute of Technology, Beijing, China; 4 Department of Emergency Medicine, Shengjing Hospital of China Medical University, Shenyang, People’s Republic of China

**Keywords:** Glioblastoma, Depressive disorder, Anxiety disorder, Bioinformatic, Patient outcome

## Abstract

**Background:**

Glioblastoma multiforme (GBM) is the most malignant tumor in human brain. Diagnosis and treatment of GBM may lead to psychological disorders such as depressive and anxiety disorders. There was no research focusing on the correlation between depressive/anxiety disorder and the outcome of GBM. Thus, the aim of this study was to investigate the possibility of depressive/anxiety disorder correlated with the outcome of GBM patients, as well as the overlapped mechanism bridge which could link depressive/anxiety disorders and GBM.

**Methods:**

Patient Health Questionnaire (PHQ-9) and Generalized Anxiety Disorder (GAD-7) were used to investigate the psychological condition of GBM patients in our department. To further explore the potential mechanism, bioinformatic methods were used to screen out genes that could be indicators of outcome in GBM, followed by gene ontology (GO) analysis, Kyoto Encyclopedia of Genes and Genomes (KEGG) analysis, and protein–protein interaction (PPI) analysis. Further, cellular experiments were conducted to evaluate the proliferation, migration capacity of primary GBM cells from the patients.

**Results:**

It was revealed that patients with higher PHQ-9 and GAD-7 scores had significantly worse prognosis than their lower-scored counterparts. Bioinformatic mining revealed that LTBP1 could be a potential genetic mechanism in both depressive/anxiety disorder and GBM. Primary GBM cells with different expression level of LTBP1 should significantly different proliferation and migration capacity. GO, KEGG analysis confirmed that extracellular matrix (ECM) was the most enriched function of LTBP1. PPI network showed the interaction of proteins altered by LTBP1. Hub genes COL1A2, COL5A1 and COL10A1, as well as mesenchymal marker CD44 and Vimentin were statistically higher expressed in LTBP1 high group; while proneural marker E-cadherin was significantly higher expressed in low LTBP1 group.

**Conclusion:**

There is closely correlation between depressive/anxiety disorders and GBM. LTBP1 could be a potential bridge linking the two diseases through the regulation of ECM.

## Background

Glioblastoma (GBM) is the most common primary WHO grade IV brain tumor in adults [1]. Tens of thousands GBM cases have been confirmed every year all over the world [2]. Moreover, GBM is also the most malignant tumor with a median survival time of only 18–24 months, and approximately 13,000 patients in the USA die of GBM each year [3], which brings a heavy burden for society, families and also, for patients themselves.

GBM patients could have significant behavioral and mood diversity and would react dramatically different when diagnosed with GBM [4]. Some of behavioral and mood negative reaction would possibly develop into psychological disorders [5]. As two of the most common types of psychological disorders, depression and anxiety had caused millions of morbidity and mortality each year all over the world [6]. It was confirmed that many biological [7, 8], genetical [9], psychological [10], and social environmental [11] factors are involved in the pathogenesis of depression and anxiety. Psychological disorders can often affect one’s physical health in a variety of known or unknown ways. For example, research had reported that depression and anxiety disorder could co-exist with a wide range of other diseases, such as cardiovascular disease [12], stroke [13], as well as many different types of cancers [14]. It seems rather intuitive that patients diagnosed with cancers would be negatively impacted by their diagnoses as well as treatments. And chronic pain caused by both cancer and the treatment could also result in emotion changes, or worse, mental disorders [15]. But the exact relationship between clinical depression/anxiety and cancers remained to be further elucidated.

It has been reported that mental disorders, especially depression and anxiety disorders, could affect the inflammation level [16], cytokines [17] and chemokines [18], neurotransmitter metabolism [19, 20], neuroendocrine function [21], and so forth, which are also recognized as possible causes for the development and heterogeneity of GBM [22]. And research have also discovered that compared with other form of cancers, glioblastoma could be concomitant with depression/anxiety [23, 24]. However, until now, there are not yet any research focusing on the possible shared molecule mechanisms between depression/anxiety and GBM.

Latent transforming growth factor-beta binding proteins (LTBPs) are large multidomain proteins that are needed for secretion, correct folding, and matrix deposition of transforming growth factor beta (TGF-β) [25]. As the first discovered protein in this family, LTBP-1 has a high expression level in several brain regions including choroid plexus, cerebral cortex, medial amygdaloid nucleus, anteromedial and midline thalamic nuclei, medial preoptic area, arcuate and dorsomedial hypothalamic nuclei, superior olive, and area postrema [26]. It has been proved by researchers that LTBP1 can regulates many neurological [27], psychological disorders [28], as well as malignant brain tumors, including malignant gliomas [29]. However, until now, there is no evidence showed that LTBP1 could function as a shared molecule mechanism that links these disorders to the prognosis of brain tumors.

In this study, we revealed that the glioblastoma patients who had higher Patient Health Questionnaire (PHQ-9) and Generalized Anxiety Disorder (GAD-7) scores could result in worse outcome after surgical treatment. To further analyze this phenomenon, a series of bioinformatic research were conducted and revealed that LTBP1 could be a crucial molecular factor for both depressive/anxiety disorder and glioblastoma. Cellular experiments showed that primary GBM cells with higher LTBP1 expression have stronger proliferation and migration capacity than those with low LTBP1 expression. In further bioinformatic mining, differentially expressed genes (DEGs) correlated with LTBP1 were screened out. Functional enrichment analysis showed the potential function and pathways of these DEGs were enriched in extracellular matrix and its components. Protein–protein interaction and hub genes showed that the collagen related genes could be the most crucial molecules that differentially expressed with LTBP1. These data provided the evidence of LTBP1 to be a potential bridge linking depressive/anxiety disorder and GBM, and laid the foundation for further therapy targeting LTBP1 for treating GBM patients who showed symptoms of depression/anxiety.

## Result

### Questionnaire revealed a negative correlation between depressive/anxiety disorder the outcome of glioblastoma patients

**Fig. 1:**
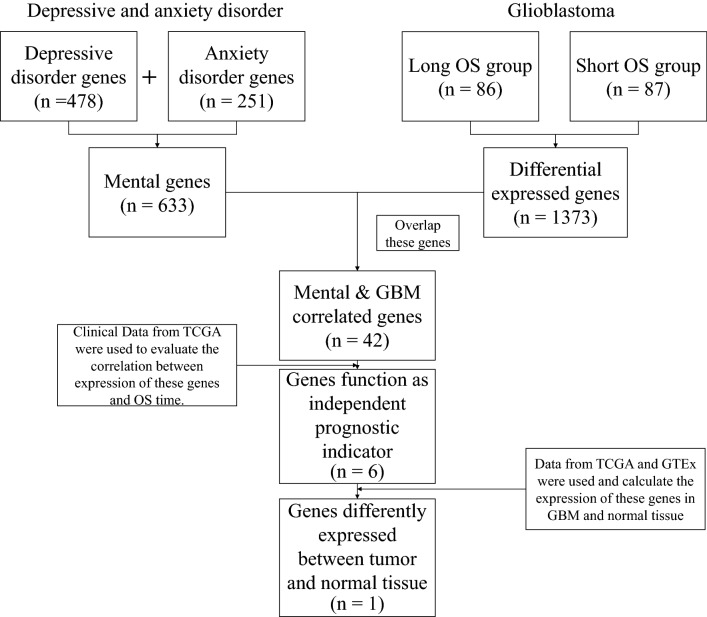
Flow chart provided the detailed step of bioinformatic mining. The flow chart showed every step of the bioinformatic mining and the basic result information for each step

Firstly, a general survey was conducted to investigate the general depressive and anxiety condition of our enrolled patients based on both Patient Health Questionnaire 9-item (PHQ-9) and Generalized Anxiety Disorder 7-item (GAD-7) scale. The detailed items of the two questionnaires were provided as Additional file 1: Table S1 and Additional file 2: Table S2. All these GBM patients were underwent brain surgery by the same group of doctors. In order to evaluate the relationship between the performance of PHQ-9/GAD-7 and the prognosis of these GBM patients, we followed up the overall survival time through telephone or chat tools. In order to further compare this relation, GBM patients were firstly divided into groups “High” and “Low”, which stand for those with higher and lower PHQ-9 or GAD-7 score than mild level, respectively; There are 19 patients who gained both higher score of PHQ-9 and GAD-7; while 25 patients gained lower scores of both the two scales **(**Additional file 3: Figure S1a). As it was showed in Fig. 1a–b, patients with higher PHQ-9 and GAD-7 scores (HIGH group) had significantly shorter overall survival time than LOW group. Cox regression analyses were used to evaluate the mortality for each group. Showed in Fig. 1c–d, GBM patients with higher PHQ-9 and GAD-7 score all presented worse mortality than their lower score counterparts. Patients with higher score in both PHQ-9 and GAD-7 presented worse outcome than that of those patients with lower scores of both PHO-9 and GAD-7(Additional file 3: Fig. S1b).

### Datasets revealed overlapped genes involving in both depressive/anxiety disorder and glioblastoma

**Fig. 2:**
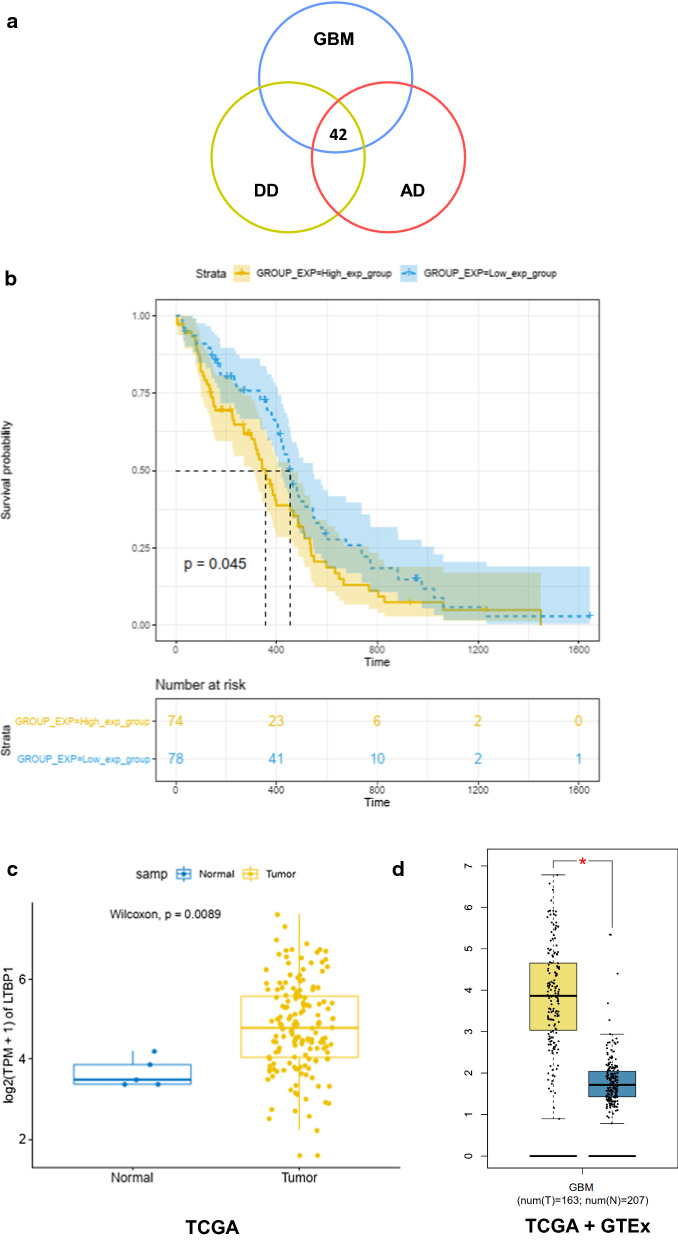
The bioinformatic results indicated that LTBP1 function as a prognostic indicator for GBM. **a** Venn diagram showed that there are 42 overlapped genes among depressive disorder, anxiety disorder and GBM. And the detailed information of genes was provided on the right side. Six genes differentially expressed of which could affect GBM patients’ prognosis were highlighted. **b** Kaplan–Meier analysis showed that high and low expression (compared with median expression) of LTBP1 can significantly influence the outcome of GBM. Log-rank (Mantel-Cox) test was used as statistical method. P = 0.045. **c–d** The expression of LTBP1 in tumor is significantly higher than it in normal tissue base on TCGA database and The Genotype-Tissue Expression (GTEx) database

In order to further investigated the potential molecular mechanisms for depressive/anxiety disorder involving in the outcome of glioblastoma, RNAseq data (173 samples) and phenotype-survival data (649 samples) with detailed survival time from the cancer genome atlas (TCGA), and total number of 633 depressive/anxiety genes (Additional file 4: Table S3) retrieved from PubMed Gene (https://www.ncbi.nlm.nih.gov/gene/) were selected to conduct bioinformatic mining. Flow chart was provided in Fig. 1 to show how the processes of bioinformatics data mining were carried out. Firstly, the RNAseq data and the phenotype data were matched and 173 samples with both RNAseq data and clinical data were obtained. These samples were then divided into high OS group (86 samples) and low OS group (87 samples) by comparing to median OS time. After this step, 1372 differentially expressed genes (DEGs) between high OS group and low OS group were screened out using ‘limma’ and ‘edgeR’ packages in R software. The DEGs and depressive/anxiety genes had 42 overlapped genes in total (Fig. 2a, Table 1). These genes were then selected for further investigation. It revealed by Kaplan–Meier analysis that LTBP1 was one of the genes which could function as a potential indicator of the GBM patients’ outcomes (Fig. 2b), together with ANKK1, FGFR1, NRG1 and BICC1(Additional file 3: Figure. S2a–b). Expression of these genes in both tumor and normal tissue were then compared. Because of the limited number of normal samples provided in TCGA database, the data from Genotype-Tissue Expression (GTEx) were also used as the data resource of normal samples. As shown in Fig. 2c–d and Additional file 3: Figure S2c, using TCGA normal data only, LTBP1 was the only gene that had significantly differential expression between normal and GBM tumor tissue. Therefore, further analysis was conducted to investigate the function of LTBP1.

**Table 1:** Genes overlapped between GBM and depressive/anxiety disorders

Gene names (42)	Resource
FKBP5; VEGFA; GDNF; PTGS2; SERPINE1; CYP2C9; MPO; TIMP1; FGFR1; CCN2; NRG1; HSPA6; PRL; CNR2; DUSP1; GNAT2; TREM1; ITGA11; NUCB2; RGS2; FMN1; MIR34C; LTBP1; AKAP12; NPY2R; FOSB; KLF11; DMRT3; GABRR1; IL1RAPL1; BICC1; ABCA13; NDST3; ESR2; CHI3L1; ADM; HTR3A; DDC; ANKK1; BACE2; SMS; LINC00578	1. https://www.ncbi.nlm.nih.gov/gene/ 2. TCGA

Total number of 42 genes were overlapped between depressive/anxiety disorders and GBM from different data resource. Among these genes, those could significantly influence the outcome of GBM were take into consideration of further researches

### Chinese Glioma Genome Atlas (CGGA) confirmed LTBP1 played a crucial role in glioblastoma

**Fig. 3:**
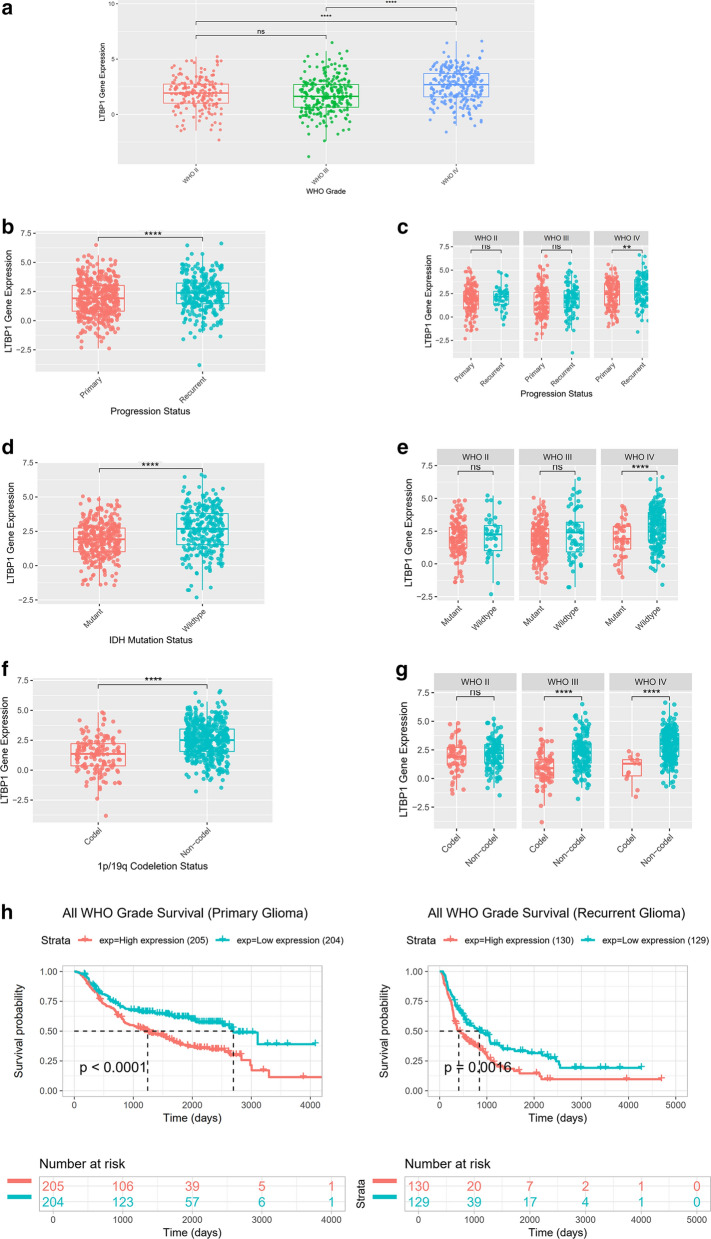
Chinese glioma genome atlas proved the evidence of the importance of LTBP1 on glioma. **a** Histogram presented the expression of LTBP1 were significantly higher in WHO IV (blue) than WHO III (green) and WHO II (red); ****P<0.0001. **b–c** The expression of LTBP1 is significantly higher in recurrent than primary gliomas in all WHO classifications. In more detail, there are significant difference of LTBP1 expression between recurrent and primary WHO IV glioma, namely GBM. No significantly difference of LTBP1 expression were observed between recurrent and primary WHO II and III gliomas. **P<0.01; ns, not significantly different. **d–e** The expression of LTBP1 is significantly higher in IDH-wildtype than IDH-mutant gliomas of all WHO classifications. In detail, there are significant expression difference of LTBP1between IDH-wildtype and IDH-mutant WHO IV glioma, namely GBM. No significantly difference of LTBP1 expression were observed between IDH-wildtype and IDH-mutant in WHO II and III gliomas. ****P< 0.0001; ns, not significantly different. **f–g** The expression of LTBP1 is significantly higher in 1p/19q Non-codeletion than 1p/19q codeletion gliomas of all WHO classifications. In detail, there are significant difference of LTBP1 expression between 1p/19q Non-codeletion and 1p/19q codeletion WHO III-IV gliomas, namely GBM. No significantly difference of LTBP1 expression were observed between 1p/19q Non-codeletion and 1p/19q codeletion in WHO II gliomas. ****P < 0.0001; ns, not significantly different. **h** Kaplan–Meier analysis showed that significantly worse outcome of GBM were observed in high LTBP1 expression group than low LTBP1 expression group for both primary and recurrent gliomas. Log-rank (Mantel-Cox) test was used as statistical method. *P*<0.0001 in primary gliomas; *P* = 0.00016 in recurrent gliomas

**Fig. 4:**
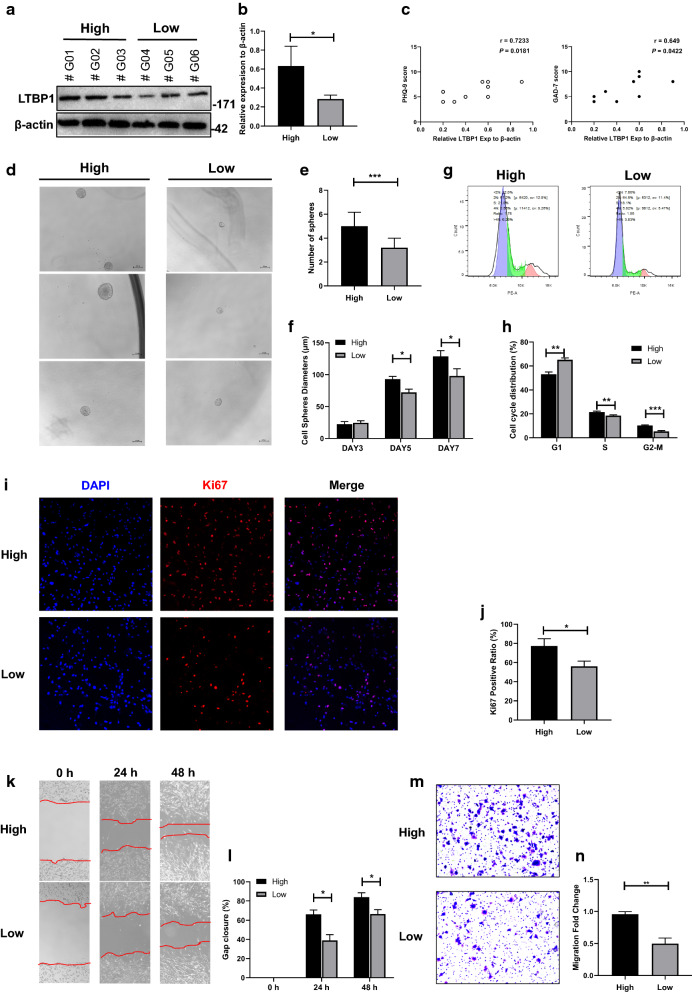
Cellular experiments showed LTBP1 could affect the function of GBM cells. **a–b** Western blotting was used to divide the primary GBM cell into high and low LTBP1 expression groups. β-actin was used as internal reference. *P<0.05; **c** The expression of LTBP1 were positively correlated with PHQ-9 and GAD-7 scores (n = 10). **d** Phase contrast pictures showed the morphology of spheres in both groups. Scale bar represented 200 mm. **e** The number of spheres in each well was counted on days 7, Data were presented as the mean ± SEM; Student’s t-test was chosen as the statistical method; n = 3, *P* = 0.0007 ***P < 0.001. **f** The diameter of spheres was measured to represent the volume. The diameters of spheres in both two groups increased gradually within 3, 5, 7 days, but the diameter of spheres in LTBP1 high expression group grew significantly faster. Data were presented as the mean ± SEM, one-way ANOVA was chosen as the statistical method. n = 3, *P < 0.05. **g–h** Cell cycle of two groups were detected by flow cytometry. A lower percentage of G1 phase, while higher percentage of S and G2-M phase were observed in high LTBP1 expression group than those in low LTBP1 expression group. **P < 0.01. ***P < 0.001. **i–j** Immunofluorescence showed more Ki-67 positive cells in high LTBP1 expression group than low LTBP1 expression group. n = 3, *P* = 0.0168. *, *P *< 0.05. **k–l** Wound healing assay showed faster migration capacity in LTBP1 high expression group than those in LTBP1 low expression group. *P < 0.05. **m–n** Transwell assay showed significantly better migration capacity in high LTBP1 group than those in Low LTBP1 group. n = 3, *P* = 0.0012

Then the data from CGGA was used to validate previous findings. The RNAseq data from CGGA contained 693 samples of glioma of different grades [30]. As showed in Fig. 3a, LTBP1 expressed significantly different among WHO II-IV gliomas: WHO IV had higher expression of LTBP1 than the other two groups, *P*<0.0001. While no significant difference of expression existed between WHO II and WHO III gliomas. And the expression of LTBP1 in primary gliomas is significantly lower than recurrent gliomas, *P*<0.001 (Fig. 3b–c). In detail, expression of LTBP1 in recurrent WHO IV glioma is significantly higher than primary WHO IV glioma, *P*<0.05. No significant expression difference of LTBP1 existed between primary and recurrent WHO II and III gliomas. Isocitrate dehydrogenase (IDH) gene mutation as well as the combined loss of the short arm chromosome 1 and the long arm of chromosome 19 (i.e. co-deletion of 1p/19q) was considered as two important genome markers that could significantly predict the outcome of gliomas. As showed in Fig. 4d–e, IDH-mutant gliomas had lower expression of LTBP1 than IDH-wildtype, *P*<0.0001. In detail, significantly different expression of LTBP1 between IDH-mutant and wildtype was existed in WHO IV but not II and III gliomas, *P*<0.0001. Similar results were also observed in 1p/19q codeletion or non-codeletion. 1p/19q codeletion gliomas had lower expression of LTBP1 than non-codeletion, *P*<0.0001. In detail, significantly different expression of LTBP1 between 1p/19q codeletion and non-codeletion was existed in WHO III and IV gliomas but not in WHO II gliomas, *P*<0.0001 (Fig. 3f–g). The Kaplan–Meier analysis that lower expression of LTBP1 have better prognosis than higher LTBP1 expression for both primary and recurrence gliomas (Fig. 3h).

### Cellular experiments proved the significant correlation of LTBP1 and the biological activity of GBM

**Fig. 5:**
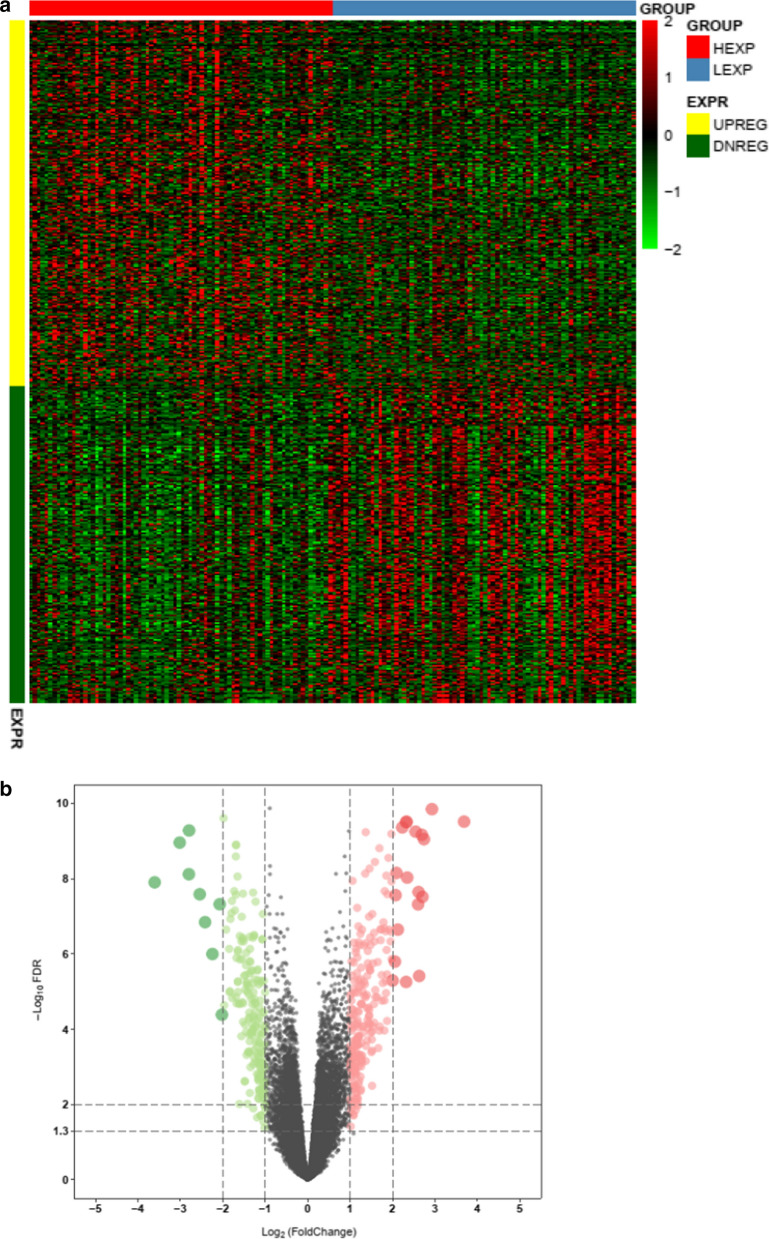
Differentially expressed genes in high and low LTBP1 expression groups. **a** Heat map showed the differential expressed genes in high and low LTBP1 expression group. **b** Volcano plot presented the upregulated (showed in red) and downregulated (showed in green) genes associated with the expression of LTBP1. FDR < 0.05 and FC >=2 were considered as statistically significant indicators. *FDR* False Discovery Rate, *FC* Fold Change

In order to confirm the function of LTBP1 on GBM at a cellular level, a series of experiments were conducted to verify the proliferation and migration ability of primary GBM cells from the patients. Firstly, these primary cells were divided into high and low LTBP1 expression group by Western blotting (Fig. 4a–b). The expression of LTBP1 was positively correlated with both PHQ-9 and GAD-7 scores (Fig. 4c). The colony formation assay was then conducted between the glioblastoma stem cells from two group. As showed in Fig. 4d–f, the number of spheres in high LTBP1 group were significantly more than that in low LTBP1 group. No significant differences in diameter were observed between the two group 3 days after the test. Then diameter of spheres in both groups gradually increased over time, but the sphere in LTBP1 high expression group increased significantly faster than those in LTBP1 low expression group. Ki-67 is considered as a marker of cell proliferation. In Fig. 4g–h, cell cycle assay showed lower percentage of G1 phase but higher percentage of S and G2-M phases in LTBP1 high expression group compared to LTBP1 low expression group. As showed in Fig. 4i–j, significantly more Ki-67 positive cells were observed in LTBP1 high expression group than those in LTBP1 low expression group. Wound healing assay was conducted to compare the migration capacity between the two groups. The gap in LTBP1 high expression group healed significantly faster than those in LTBP1 low expression group (Fig. 5k–l). In Fig. 4m–n, Transwell assay was used to further evaluate the migration capacity between high and low LTBP1 expression group. Significantly better migration capacity was observed in cells of high LTBP1 group than those of low LTBP1 group.

### The pathways potentially regulated by the differentially expression of LTBP1

**Fig. 6:**
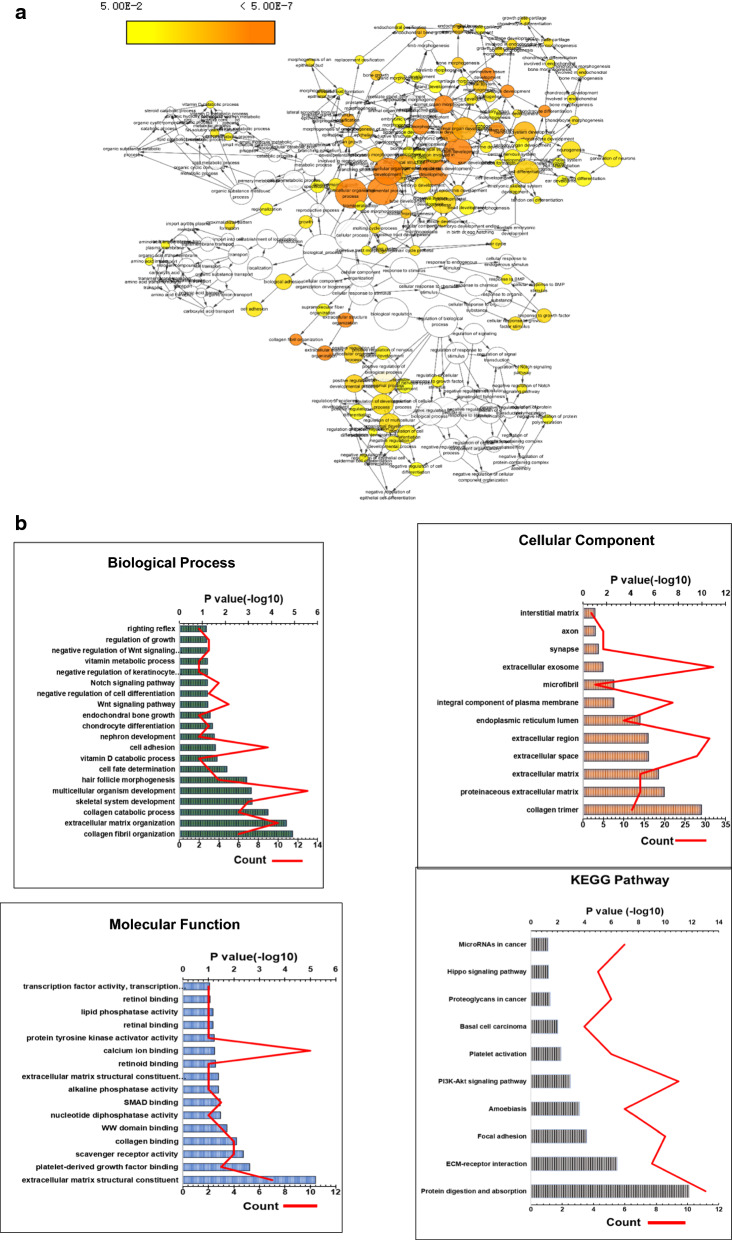
Gene ontology analysis **a** binGO plots showed the most significantly enriched function and pathways regulated by differentially expression of LTBP1. The adjust P value was used as statistically significant indicators. The shade of color is used to show the significance of the level. The top 10 of the function and pathways were ‘collagen fibril organization’, ‘multicellular organism development’, ‘developmental process’, ‘system development’, ‘multicellular organismal process’, ‘extracellular matrix organization’, ‘extracellular structure organization’, ‘animal organ morphogenesis’, ‘connective tissue development’ and ‘anatomical structure development’. **b** The combined charts showed the top 20 (or total) pathways and functions of Biological Process, Cellular Components, Molecular Functions and KEGG pathways regulated by the differentially expression of LTBP1. The column stands for the -Log10(P value), and the red line stands for the counts of genes involved in the pathways

Heatmap (Fig. 5a) and volcano plot (Fig. 5b) showed that there were 22493 DEGs between LTBP1 high expressed group and LTBP1 low expressed group (adjusted p value < 0.05) and the numbers of genes upregulated and downregulated were 11114 and 11139, respectively (Additional file 5: Table S4). The top 20 up and downregulated genes were provided as Table 2. In order to analyze the function of LTBP1 on the tumor biology of gliomas, function enrichment analysis was conducted using the top 150 up and down regulated DEGs. The total landscape of significantly changed Gene Ontology (GO) terms were conducted with binGO and showed that ‘developmental process’, ‘collagen fibril organization’, ‘multicellular organism development’, ‘system development’, ‘multicellular organismal process’, ‘extracellular structure organization’, ‘extracellular matrix organization’, ‘animal organ morphogenesis’, ‘connective tissue development’, ‘anatomical structure development’ were the top ten process (Fig. 6a). In a more detailed way, biological processes (BP), molecular functions (MF) and cellular components (CC) in GO enrichments as well as Kyoto Encyclopedia of Genes and Genomes (KEGG) pathway were conducted with the Database for Annotation, Visualization and Integrated Discovery (DAVID) database. As showed in Fig. 6b, the top BP enrichments were ‘collagen fibril organization’, ‘extracellular matrix organization’, ‘collagen catabolic process’, ‘skeleton system development’, ‘multicellular organism development’, ‘cell fate determination’, etc. The top CC enrichments were ‘collagen trimer’, ‘proteinaceous extracellular matrix’, ‘extracellular matrix’, ‘extracellular space’ and ‘extra cellular region’, etc. The top MF enrichments were ‘extracellular matrix structural constituent’, ‘platelet-derived growth factor binding’, ‘scavenger receptor activity’, ‘collagen binding’ and ‘WW domain binding’, etc. The top KEGG pathways were ‘protein digestion and absorption’, ‘ECM receptor interaction’, ‘Focal adhesion’, ‘amoebiasis’ and ‘PI3K-Akt signaling pathway’, etc.

**Table 2:** Top 20 upregulated and downregulated genes

Upregulated genes		Downregulated genes	
Gene name	Fold change (log2FC)	Gene name	Fold change (log2FC)
CAPN6	5.705223	SEPTIN14	− 3.605153078
RHBG	4.191004	KLRC2	− 2.788656382
LINC01445	3.68606	SNORD17	− 2.594281617
PRG4	3.37865	GLRA3	− 2.543376544
DNM3OS	3.374032	HIST1H1E	− 2.466989188
TNMD	2.928818	TMPRSS2	− 2.41745684
ALDH1A3	2.785768	7SK	− 2.404196134
LINC01139	2.770565	SNORA73B	− 2.360291834
HMGA2	2.739822	EGFR	− 2.243517088
IGF2BP1	2.70558	CA10	− 2.072284579
EDN3	2.691307	RN7SL2	− 1.981428914
CILP	2.66492	HMX1	− 1.967819273
DLK1	2.627985	COL20A1	− 1.922927023
DPT	2.613928	PERM1	− 1.849366765
SCARA5	2.602058	GPR17	− 1.837058102
HES2	2.585225	TPTEP1	− 1.834874915
CYP24A1	2.542172	MIR219A2	− 1.82019412
COL6A3	2.346062	RN7SL3	− 1.795601021
CTLA4	2.330009	PCDH11X	− 1.778882755
PRAME	2.320485	LINC02367	− 1.694275084
LINC01206	2.194436	SLC29A1	− 1.692683409

The top up and downregulated genes regulated by LTBP1 and the fold change calculated by log2FC method

### Protein–protein interaction (PPI) network construction

**Fig. 7:**
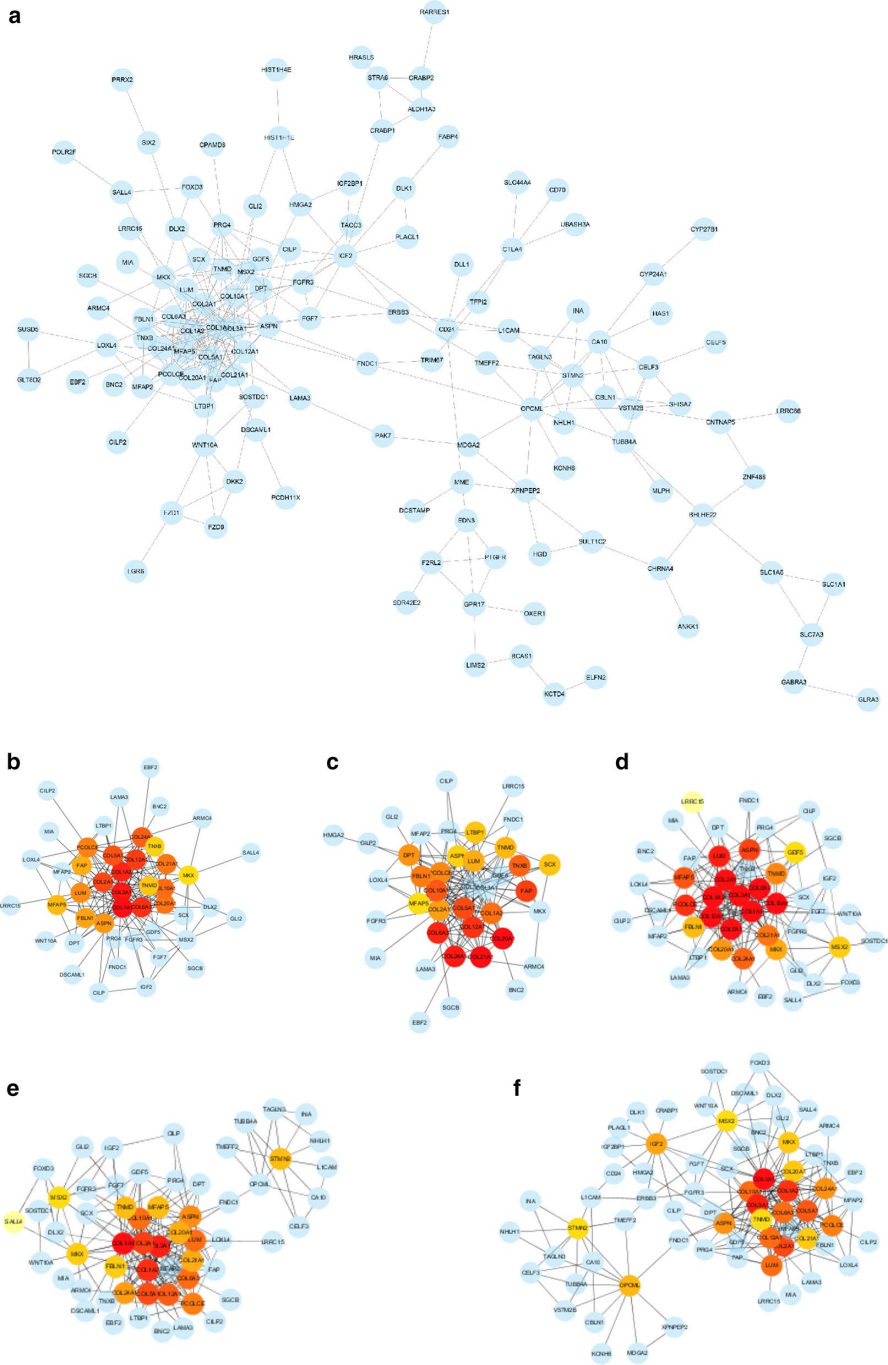
Protein-protein interactions (PPI) related to LTBP1. **a** The PPI of the top 150 of upregulated and down regulated genes in DEGs. **b–f**. Hub genes calculated by 5 methods in CytoHubber

**Fig. 8:**
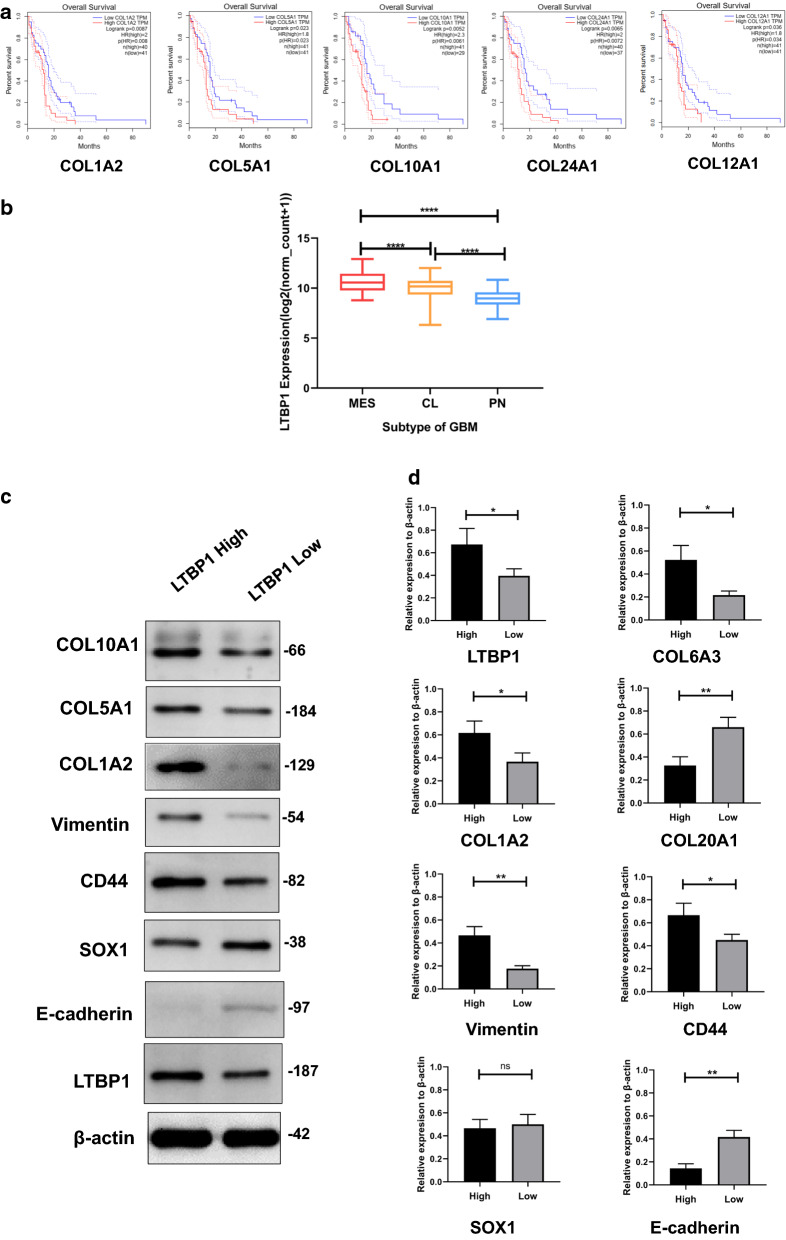
The correlation of LTBP1 to the outcome of GBM patients. **a** Kaplan–Meier plots showed that the expression difference of hub genes COL1A2, COL5A1, COL10A1, COL12A1 and COL24A1 significantly influenced the prognosis of GBM based on TCGA data. **b** LTBP1 were differentially expressed among subtype of GBM, namely mesenchymal (MES), classical (CL) and proneural (PN). **c** Western blotting revealed the expression of hub genes and mesenchymal, proneural markers were differentially expressed between high and low LTBP1 group

PPI networks were conducted with top of the up- and downregulated genes using STRING database. Ultimately, 129 nodes and 307 edges were established (Fig. 7a). We then used the ranking method ‘MCC’, ‘DMNC’, ‘EPC’, ‘MNC’, and ‘degree’ in cytoHubba to select hub genes. As showed in Fig. 7b–f, the top 20 of hub genes were calculated by each method. In order to confirm the most important hub genes among all, the hub genes calculated with 5 ranking methods were overlapped, and 13 genes were obtained (Table 3), namely COL1A2, COL2A1, COL5A1, COL12A1, COL10A1, COL6A3, LUM, PCOLCE, SPN, COL24A1, COL20A1, COL21A1, TNMD. In order to further confirm the exact expression of these hub genes, western blotting was conducted. Among these genes, the expression of COL1A2, COL5A1, COL10A1, COL24A1 and COL12A1 could significantly affect the prognosis of GBM patients, while other genes did not function as indicator of prognosis (Fig. 8a, Additional file 3: Figure S3). Western blotting revealed that expression of COL1A2, COL5A1, COL10A1 were significantly different between high and low LTBP1 group (Fig. 8c).

**Table 3:** Hub genes with different methods

MCC	Score	DMNC	Score	EPC	Score	Degree	Score	MNC	Score	Overlapped
*COL1A1*	Score	*COL20A1*	0.897868	*COL2A1*	61.909	*COL1A1*	30	*COL1A1*	28	*COL1A2*
*COL3A1*	3716032	*COL21A1*	0.897868	*COL5A1*	61.909	*COL3A1*	24	*COL3A1*	24	*COL2A1*
*COL1A2*	3715972	*COL24A1*	0.88234	*COL6A3*	61.909	*COL1A2*	20	*COL1A2*	19	*COL5A1*
*COL6A3*	3715873	*COL6A3*	0.78828	*COL3A1*	61.909	*COL2A1*	19	*COL2A1*	18	*COL12A1*
*COL12A1*	3715201	*COL12A1*	0.711055	*COL1A1*	61.909	*COL5A1*	16	*COL5A1*	16	*COL10A1*
*COL2A1*	3714722	*FAP*	0.665688	*COL1A2*	61.909	*COL10A1*	15	*COL12A1*	15	*COL6A3*
*COL5A1*	3714653	*COL5A1*	0.646143	*COL12A1*	61.868	*COL12A1*	15	*COL10A1*	14	*LUM*
*COL24A1*	3710306	*COL10A1*	0.641885	*COL24A1*	61.845	*COL6A3*	15	*COL6A3*	14	*PCOLCE*
*COL10A1*	3669122	*TNXB*	0.618139	*ASPN*	61.839	*LUM*	15	*LUM*	14	*ASPN*
*COL21A1*	3633873	*COL1A2*	0.616463	*LUM*	61.8	*PCOLCE*	14	*PCOLCE*	13	*COL24A1*
*COL20A1*	3628800	*FBLN1*	0.612303	*PCOLCE*	61.754	*ASPN*	13	*ASPN*	12	*COL20A1*
*PCOLCE*	3628800	*PCOLCE*	0.587563	*COL20A1*	61.702	*COL24A1*	13	*COL24A1*	11	*COL21A1*
*LUM*	80795	*DPT*	0.583436	*COL10A1*	61.641	*IGF2*	12	*COL20A1*	10	*TNMD*
*ASPN*	41431	*COL2A1*	0.580315	*MFAP5*	61.501	*OPCML*	11	*COL21A1*	10	
*FBLN1*	5235	*LUM*	0.563057	*COL21A1*	61.432	*MKX*	10	*STMN2*	9	
*FAP*	846	*TNMD*	0.525062	*TNMD*	61.391	*COL20A1*	10	*TNMD*	9	
*MFAP5*	240	*LTBP1*	0.51861	*MKX*	61.365	*COL21A1*	10	*MFAP5*	9	
*TNMD*	200	*SCX*	0.51861	*FBLN1*	61.359	*MSX2*	9	*MKX*	8	
*TNXB*	192	*ASPN*	0.512224	*GDF5*	60.695	*STMN2*	9	*FBLN1*	8	
*MKX*	144	*MFAP5*	0.477329	*MSX2*	60.232	*TNMD*	9	*MSX2*	7	

Hub genes and scores calculated with five different method in Cytohubber APP in Cytoscape, as well as the overlapped hub genes

### Subtype distribution difference between high and low LTBP1 group

It had been reported that alternation of ECM could result in initiation of epithelial mesenchymal transition of tumor [31]. In order to confirm it is also the mechanism with which ECM altered by LTBP1 could enhance the proliferation and migration capacity in GBM, we firstly investigate the subtype date of GBM from TCGA and conduct a correlation analysis between high and low group. As showed in Fig. 8b, the expression of LTBP1 were significantly higher in mesenchymal subtype (MES) than any other two groups. And the proneural subtype had the lowest expression of LTBP1. To confirm this finding, western blotting was conducted to evaluate the expression of mesenchymal marker Vimentin and CD44, and proneural subtype marker E-cadherin were significantly higher in LTBP1 high and low expression group, respectively. Proneural marker SOX1 did not show significant expression difference between the two groups. (Figure 8c).

## Discussion

Being diagnosed with a tumor and underwent surgery, especially brain surgery, may bring major negative impact on one’s life. Some of the patients might just meet transient or mild negative mood about the diagnosis or treatment, but some may suffer persistent negative mood in a moderate or severe level, could then develop into a multifaceted anhedonic state, and impaired cognitive function, which could be defined as depressive/anxiety disorders [24]. It had been showed by many research the certain correlation of tumor with depressive/anxiety disorders, and some of these research are focusing on the influence of depressive/anxiety on the outcome of tumor patients [32]. But none of these researches provided any information about the potential mechanism of depression/anxiety disorder on the prognosis of tumor patients. Measured with the widely used scale PHQ-9 and GAD-7 questionnaires, we firstly screened out the psychiatric condition of 73 GBM patients in our department. We found that those GBM patients with higher PHQ-9 and GAD-7 score have significantly worse prognosis than their lower scored counterparts. It seems the case that glioblastoma and depressive/anxiety disorder share some common etiological factors, such as inflammation [33, 34], neurotransmitter metabolism [35], glutamine [36, 37], neural plasticity [38, 39], etc. Nevertheless, none of these factors were confirmed by further research. With the rapid development of human genome project and the cancer genome atlas system, it is now possible for us to start a genetic-level analysis to evaluate genetic alternations across diseases. As diseases with multiple genetic abnormalities, there should be a possibility that there are some genetic alternations overlapped in the brain which may not only result in susceptibility to depression and anxiety, but also in the malignant progression of GBM which result in worse prognosis. As a result, the bioinformatic mining were conducted.

Using a series of bioinformatic methods, six genes related to depressive/anxiety disorders which have negative impact on the prognosis of GBM were screened out. And among these genes, LTBP1 was differentially expressed between tumor and normal tissue. LTBP1, as an important member of the latent TGF-β1-binding protein family, has been shown to bind to the latent form of TGF-β and escort it during secretion, thus enhancing TGF-β bioavailability [25]. Researchers have found that LTBP1 could play a pivotal role during the processes of psychomotor retardance and disorders [40]. And LTBP1 and its target TGF-β1 were also involved in Alzheimer disease and depression [28]. On the other side, it was also reported that LTBP1 was closely involved in the processes of glioma [29, 41]. However, there is neither evidence that can provide the exact mechanism of LTBP1 on depressive/anxiety disorder as well as GBM, nor any research focusing on the connection linking depressive/anxiety disorders and the prognosis of GBM. We discovered that the expression of LTBP1 was positively correlated with the PHQ-9 and GAD-7 score among these GBM patients. This encouraged us to further evaluate the function of LTBP1 and possibly find a potential bridge built by LTBP1 that could link depressive/anxiety and GBM.

We firstly confirmed the negative influence of LTBP1 on the outcome of gliomas with the data from Chinese Glioma Genome Atlas. We then found that the primary GBM cell with higher expression of LTBP1 have stronger proliferation and migration capacity than those with lower expression of LTBP1. These results indicated the direct function of LTBP1 on GBM. We further conducted the functional enrichment analysis and the protein–protein interaction of the differentially expressed genes regulated by LTBP1. To our astonishment, the findings revealed that the enriched functions and pathways were mostly related to extracellular matrix (ECM). And hub genes closely related to collagen formation and ECM, such as COL1A2, COL10A, etc. which were also key indicators on GBM outcome, were also differentially expressed between high and low PHQ-9 and GAD-7 groups in our research.

Extracellular matrix seems to be a probable mechanism by which we could link the depressive/anxiety disorder and worse outcome of GBM. In terms of tumor, including GBM, the ECM regulates tissue development and homeostasis, and its dysregulation contributes to neoplastic progression [42]. The cancer-associated ECM is not only an integral feature of a tumor but also actively contributes to its histopathology and behavior [43]. Research had discovered that ECM-associated alternations could overwhelmingly influence the capabilities of human tumors including sustained proliferation [44], evasion of growth suppression [31], death resistance [45], replicative immortality [46], induced angiogenesis [47], initiation of invasion [48], dysregulation of cellular energetics [49], avoidance of immune destruction [50] and chronic inflammation [51]. During the tumorigenesis and development gliomas, significant alterations of the ECM in brain tissues could be triggered by glioma tissue. Such changes include altered synthesis of the components by the tumor cells, extensive degradation of the ECM at the invasive front of the tumor, as well as an elevated level of synthesis of ECM components by normal tissues in the vicinity of the invading tumor. Moreover, it was revealed that glioma cells had the ability to actively migrate using blood vessels or axons as guide paths due to interaction with the ECM. Additionally, glioma cells can secrete their own ECM components, including HA, brevikan, tenascin C and thrombospondin, as well as fibronectin, which are actively expressed in the ECM of the developing nervous system along cell migration paths. [52, 53]. This could partly explain that the in vitro GBM cell could also proliferate significantly different in our experiment. On the other hand, the function of ECM was also significant factor on the process of depressive/anxiety disorders. It is discovered that ECM alterations in the cognitive component was associated with depressive-like behavior [54, 55]. And ECM markers such as MMP9 and sICAM1 were also reported to deficit cognitive function in human brain which may result in serious psychiatric disorders such as depressive disorders and bipolar disorders [56].

**Fig. 9:**
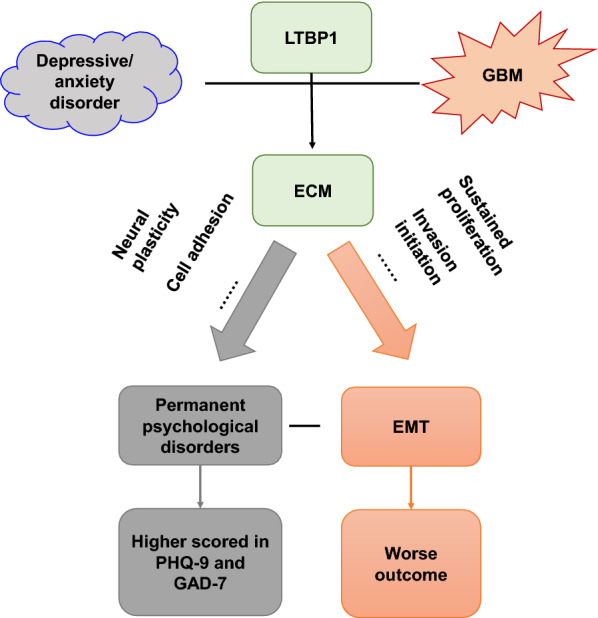
Schematic diagram showed the potential mechanism of depression affecting the outcome of GBM by LTBP1

To our knowledge, there are many possibilities that the alternation of ECM by differentially expression of LTBP1 in brain may link depressive/anxiety disorders and glioblastoma (Fig. 9). Firstly, brain neural tissue functions as a dynamic network-beneficial synaptic connections need to be maintained, and other reconstructed to match changing input stimuli. Cell–cell interactions in the brain, similarly to other tissues, are based on direct contacts via cadherins and signaling receptors, as well as cell–matrix interactions with the ECM [57]. Differentially expressed LTBP1 could change the components of ECM and stabilization of network, alter the neural plasticity and change the adhesion and interaction among of brain, which may result in susceptibility of neurological and psychological disorders, including depressive/anxiety disorders. Similarly, such alternation could also trigger the aggressiveness and migration of GBM cells and result in worse prognosis [58]. Secondly, LTBP-1 targets latent TGF-β1 to the ECM by interacting with different proteins including fibronectin and fibrillin, generating deposits of latent TGF-β1 accessible for cell-mediated activation and regulating cancer cell proliferation and immunity [59]. Immunology homeostasis instabilities may cause deficits of cognitive and memory in human brain, which could be a potential cause of depressive and anxiety disorders [60]. On the other side, as important component of neural microenvironment, ECM could regulate the neural inflammation in central nervous system [61]. Inflammation is believed to be a typical pathology changes in depressive and anxiety disorder [62], and chronic inflammation may also result in the epithelial mesenchymal transformation (EMT) of glioblastoma, and in turn cause worse outcome in GBM patients [63]. The changing of ECM by LTBP1 could function as an indicator of depressive/anxiety disorder and GBM by regulating inflammatory responses. And in order to confirm the case, we evaluated the expression of epithelial and mesenchymal markers in high and low LTBP1 group and found a higher expression of mesenchymal markers such as vimentin and CD44, as well as lower expression of typical epithelial markers such as E-cadherin, in high LTBP1 group. Moreover, flow cytometry cell cycle assay revealed a higher proportion of S and G2-M phases in LTBP1 high expression group, which was reported by many other researches to be an important feature of EMT in many type of cancers, including GBM [64–66]. As for the reason why no expression differences of SOX1 was observed between the two group, we suppose that SOX1 is a transcription factor that expressed almost in all tissue and cells with high proliferative rate and stemness. All subtype of GBM have a relatively high expression of SOX1, so no significantly despite slightly difference could be observed between high and low LTBP1 groups. But these findings are enough to prove the evidences that there were more cells classified as mesenchymal subgroup between primary GBM cells from high and low LTBP1 groups, namely those with high and low PHQ-9 and GAD-7 scores, which may result in the significant difference of proliferation and migration rate. However, due to the lack of animal model that could mimic both depressive/anxiety as well as GBM, we could not provide any in vivo experiments that directly prove the evidence of the interaction between depressive/anxiety disorders and GBM. Our speculation was based on only bioinformatic and cellular experimental results, and further research were still needed to elucidate the exact mechanism of this phenomenon.

There were also many drawbacks in the research. Firstly, due to the different educational background of these patients, we could not provide a systematically investigation of the psychological condition of GBM patients in our department, with a complete and detailed scales or tools. Secondly, due to the limitation of the small sample size, we cannot perform high-throughput sequencing or single-cell sequencing of large samples to verify our data mining results. In the future studies, we would conduct more comprehensive researches to make up for the current shortcomings.

## Conclusion

To sum up, in this study, we revealed a directed correlation between depressive/anxiety disorder and the outcome of GBM. By a series of bioinformatic methods, we found that LTBP1 could be a bridge which link depressive/anxiety disorder and GBM. And the potential mechanism of LTBP1 on both depressive/anxiety disorder and GBM could be result from the regulation of ECM in the brain. Therefore, the psychological condition of GBM patients should be taken into consideration in our future clinical work, and the targeted therapy towards LTBP1 might be a promising research direction for our future clinical and experimental research, which may further provide us with a shared mechanism for both GBM and depressive/anxiety disorders, as well as a possible precise treatment for GBM patients showed the symptom of depression and anxiety disorders.

## Material and method

### Subjects

A total number of 73 patients (33 males and 40 females), aging from 32 to 68 (43.63 ± 0.91) years old with pathological diagnosis of GBM were enrolled in our project. All the enrolled GBM patients should have KPS score higher than 70. This study was approved by the local Ethics Committee and was conformed to the principles outlined in Declaration of Helsinki. Written informed consent was provided by all patients.

### Instruments

In order to meet the different education background of all ages, Patient Health Questionnaire 9 items (PHQ-9) and Generalized Anxiety Disorder 7-item (GAD-7) scale were chosen as our measurement for major depressive disorder and anxiety disorder, both of which are widely used as a reliable measurement to evaluate one’s depressive or anxiety condition. All the patients were asked to complete the two questionnaires after they were diagnosed with glioma (even though not yet with a certain diagnosis of glioblastoma before verified with pathology methods). Scored with higher and lower than median were divided into high and low PHQ-9 and GAD-7 group. The detail items of PHQ-9 and GAD-7 were presented as Additional file 1: Table S1 and Additional file 2: Table S2.

### Database

RNA expression for Glioblastoma Multiforme using TCGA-GBM-HTseq (07-19-2019) was obtained from the GDC TCGA data portal (https://docs.gdc.cancer.gov), Clinical data such as gender, age, histological type, survival and outcome were also downloaded from TCGA data portal. Depressive and anxiety disorder related genes were retrieved from PubMed Gene (https://www.ncbi.nlm.nih.gov/gene/) using key words ‘depression’, ‘depressive disorder’, ‘major depressive disorder’, ‘anxiety’ and ‘anxiety disorder’. For validation, mRNA sequencing data (mRNAseq_693) for GBM patients were obtained from the Chinese Glioma Genome Atlas (CGGA) data portal (http://www.cgga.org.cn/). Clinical data of survival and outcome were also downloaded from the CGGA data portal.

### Differentially expressed genes

The FPKM data downloaded from GDC data portal (HTseq-FPKM) was used to mining the differentially expressed genes between high and low expression of LTBP1. Differential expression analysis of two conditions was performed using the edgeR R package (3.9.1); Corrected *P* value of 0.05 and absolute fold change of 2 were set as the threshold for significantly differential expression. Samples were divided into high and low LTBP1 by median level of expression.

### Culture of primary glioblastoma cells

Briefly, jelly-like tumor tissue was obtained during surgery, and removed into a sterilized 50 mL centrifuge tube with ice-cold PBS in it. Then the tumor tissue was carefully transported from operating room to laboratory in an icebox. Discard the supernatant, place the tissue sample in a sterile dish, and cut it into 1 mm^3^ pieces with sterilize scissors and tweezers. Then collect the cut sample into a 15 ml centrifuge tube, add PBS with 1% penicillin–streptomycin, mix and shake up and down for three times in order to remove the remaining red blood cells as thoroughly as possible. After the upper layer of liquid is clear, carefully remove the supernatant, add about 3 ml trypsin for every 2 cm^3^ tissue, incubate at 37 °C for 10 min, and shake it every 2 min to make the tissue fully digested. After the digestion, the upper fluid would be turbid, let the tube stand for 2 min, then move the supernatant into an Eppendorf (EP) tube. Centrifuge the EP tub for 5 min with 1000 rpm, and put it in a culture disk with Dulbecco’s Modified Eagle Medium with 10% fetal bovine serum. Incubate under a temperature of 37 °C and 5% CO_2_. Medium was changed every 2 days. Primary GBM cells were irregular spindle-like cell under microscope. Primary GBM stem cells were cultured in stem cell culture media DMEM or neurobasal media (Life Technologies Corporation) supplemented with 20 ng/mL FGF-2 (PeproTech), 20 ng/mL EGF (PeproTech), B27 and N2 supplemental factors (Gibco) and antibiotics (penicillin and streptomycin). Isolated GSCs grew as sphere after 1 to 2 months of continuous culturing. GSCs were tested for their capability to self-renew using the sphere-formation assay described below.

### Sphere formation assay

In summary, GBM spheres were harvested and dissociated with 0.25% trypsin. After centrifugation, the cell suspensions of expanded glioblastoma cells were seeded in a 96-well plate with 3–4 cells in each well. After incubation for 3, 5 and 7 days in GSC proliferation medium, the long diameter and shape of spheres were measured. The number of GSC spheres were counted. The fixed area (10 mm^2^) at the center of each well was converted into a digital image using a digital still camera (Axio Observer A1), and the number of spheres was counted by Image-Pro Plus 6.0 (Media Cybernetics).

### Immunofluorescence

In short, the cells were plated in 12-well culture plates with cover glass pre-coated with 10% polylysine. Cells were cultured for 3 days then fixed with 4% paraformaldehyde (PFA) in PBS, permeabilized with 0.1% Triton-X100 and blocked with 5% normal goat serum in PBS. Then, they were incubated overnight at 4 °C with: Rabbit anti-Ki-67, 1:200 (Abcam). After washing with PBS, cells were incubated with Alexa Fluor^®^555 anti-Rabbit IgG, 1:4000 (Abcam) for 1 h at room temperature. Cells were then counter-stained with DAPI (Southern Biotechs). The images were captured using FLUOVIEW FV 10i (OLIMPUS).

### Flow cytometry analysis for cell cycle

1 × 10^6^ cells were seeded into 6-well plates. After 36 h, cells were collected and fixed in chilled 70% ethanol at −20 °C for 2 h, followed by washing with ice-cold phosphate-buffered saline (PBS), and the fixed cells were stained with 50 g/ml propidium iodide (PI) in darkness at room temperature for 30 min before analysis. The samples were then analyzed with BD LSR Fortessa (BD Biosciences).

### Cell migration and invasion assay

The migration assay was performed with transwell insert chambers (8 μm pore size, Corning, USA). In short, about 2 x 10^4^ cells were seeded into the upper chamber in serum free medium in triplicate. The lower chamber was filled with 600 μl DMEM medium containing 10% FBS (chemo-attractant). After incubation for 24 h, cells in upper chambers (namely non-migrating cells) were removed by cotton swab, and cells migrating to the lower surface of membrane were fixed using methanol and stained with 0.1% crystal violet. The migrating cells were counted at least 10 visual fields per membrane under the light microscope.

### Functional and pathway enrichment analysis

The Database for Annotation, Visualization, and Integrated Discovery (DAVID, https://david.ncifcrf.gov/) was used to perform Gene Ontology (GO) enrichment analysis. DAVID is an online tool for systematic and integrative annotation and enrichment analysis that can be used to reveal biological meaning related to large gene lists. GO analysis for the cellular component, biological process (BP), and molecular function (MF) categories and Kyoto Encyclopedia of Genes and Genomes (KEGG) pathway enrichment analysis were performed for the selected genes using the DAVID. A p value < 0.01 was considered statistically significant. BinGO app from Cytoscape were using to visualize the functional enrichment of differentially expressed genes.

### Protein–protein interaction (PPI) network generation and hub gene analysis

The Search Tool for the Retrieval of Interacting Genes (STRING) is an online database used to predict PPIs, which are essential for interpreting the molecular mechanisms of key cellular activities in carcinogenesis. In this study, we used the STRING database to build a PPI network of differentially expressed genes between high and low LTBP1 group. The cut-off standard was defined as an interaction score of 0.4 (medium). The target hub genes used for further analysis they were in the top 20 genes according to 5 cytoHubba ranking methods using Cytoscape software. The cytoHubba plug-in explore important nodes/hubs and fragile motifs in an interactome network by several topological algorithms including Degree, Edge Percolated Component (EPC), Maximum Neighborhood Component (MNC), Density of Maximum Neighborhood Component (DMNC), Maximal Clique Centrality (MCC) and centralities based on shortest paths, such as Bottleneck (BN), EcCentricity, Closeness, Radiality [67]. In general, proteins with a high “degree” are more likely to be key proteins, and MCC has better predictions for key proteins in the yeast interaction network. The protein nodes are ranked in order of importance. The darker the color, the more important the protein is in the interaction network. The hub genes were then screened out with the intersection of these different methods.

### Western blot

Western blot assay was performed using primary GBM cells. 50 mg of total protein in each group were separated on 10% SDS-PAGE, then transferred to a 0.22 mm PVDF membrane (Millipore). The membranes were blocked with 5% skimmed milk at room temperature for 2 h, and then incubated with specific primary antibodies at 4 °C overnight. The membranes were incubated with appropriate HRP-conjugated secondary antibodies diluted at 1:5000 (Boster) at 37 °C for 1 h. Protein bands on the membrane were visualized with ECL Kit (Millipore) using FluorChem FC system (Alpha Innotech Corporation).

### Wound healing assay

In short, a marker pen was used to draw a straight line on the back of the 6-well plate with a ruler, and evenly draw a horizontal line, about every 0.5 ~ 1 cm, across the hole. Each hole crosses at least 5 lines. Add about 5 x10^5^ cells in each well, cultured overnight. On the second day, scratch straight horizontal lines on the surface of the cultured cells with 200μL pipette head. Wash the cells 3 times with PBS, remove the scratched cells, and add serum-free medium. Take pictures of the 6-well plates as baseline. Cultured the plates in incubator with 37 °C and 5% CO_2_. 12 and 24 h later, take out the plates and take pictures. The gap among different groups were measured.

### Statistical tests

All variables of interest are presented as the mean ± standard error and analyzed through Student’s t-test, since variables had normal distribution; and one-way ANOVA using GraphPad Prism software (Version 5.01; GraphPad Software, Inc). P < 0.05 was considered as a standard of statistically significant.

## Supplementary information

**Additional file 1: Table S1.** Detail items and severity of two surveys.

**Additional file 2: Table S2**. The severity distribution of PHQ-9 and GAD-7 in enrolled GBM patients.

**Additional file 3: Figure S1.** Overlapped results of PHQ-9 and GAD-7 among GBM patients. **a**. Pie charts revealed that 36 and 37 patients gained high and low PHQ-9 scores; while 32 and 41 patients gained high and low GAD-7 scores. 19 and 25 patients gained both high and both low PHQ-9 and GAD-7 scores. **b**. Kaplan Meier plot showed that those patients with both higher PHQ-9 and GAD-7 scores (referred as High-scored) had significantly worse outcome than those with both lower PHQ-9 and GAD-7 scores (*P* = 0.0005). **Figure S2.** Bioinformatic mining screened out six potential genes which is involved in both depressive/anxiety disorders and GBM. **a** Significant difference of outcome could be observed between high and low expression of these six genes. Log-rank (Mantel-Cox) test was used as statistical methods. *P* ^ANKK1^= 0.016, *P* ^FGFR1^ = 0.042, *P* ^NRG1^= 0.0062, *P* ^BICC1^= 0.039 **b** Heatmap and volcano plot shows the differentially expressed genes between high and low expression of the six genes. **c** The expression of these six genes between tumor and normal tissue revealed significantly different expression of LTBP1 in GBM tissue than that in normal tissue. Wilcoxon test was used as statistical methods. *P* ^ANKK1^= 0.029 (higher expression in normal tissue), *P* ^FGFR1^ = 0.37, *P* ^NRG1^= 0.69, *P* ^BICC1^= 0.29. **Figure S3.** The hub genes differential expression of which did not influence the outcome of GBM. The hub genes COLA3, LUM, COL2A1, PCOLCE, COL21A1, COL20A1, ASPN and TNMD were not a significant indicator of the outcome in GBM patients. **Figure S4** Protein-protein interactions (PPI) related to LTBP1 screened out with only “experimental evidence” selected in STRING database.

**Additional file 4: Table S3.** Differentially expressed genes between high and low LTBP1 expression group.

**Additional file 5: Table S4.** The overlapped genes of depressive and anxiety disorders.

## Data Availability

All data generated or analyzed during this study were included in this published article and its additional files.

## Abbreviations

GBM: Glioblastoma multiforme
PHQ-9: Patient Health Questionnaire 9-item
GAD-7: Generalized Anxiety Disorder 7-item
TGF-β: Transforming Growth Factor β
LTBP1: Latent transforming growth factor beta binding protein 1
TCGA: The cancer genome atlas
CGGA: Chinese glioma genome atlas
GO: Gene Ontology
BP: Biological process
MF: Molecular function
KEGG: Kyoto Encyclopedia of Genes and Genomes
PPI: Protein–protein interaction
STRING: The Search Tool for the Retrieval of Interacting Genes
GSC: Glioma stem cell. ECM: Extracellular matrix
