# Correction to: LTBP1 plays a potential bridge between depressive disorder and glioblastoma

**DOI:** 10.1186/s12967-020-02619-y

**Published:** 2020-11-27

**Authors:** Xiaojun Fu, Pei Zhang, Hongwang Song, Chenxing Wu, Shengzhen Li, Shouwei Li, Changxiang Yan

**Affiliations:** 1 Department of Neurosurgery, Sanbo Brain Hospital, Capital Medical University, Xiangshanyikesong 50#, HaiDian District, Beijing, 100093 China; 2 Capital Medical University, Beijing, People’s Republic of China; 3 Beijing Institute of Technology, Beijing, China; 4 Department of Emergency Medicine, Shengjing Hospital of China Medical University, Shenyang, People’s Republic of China

## Correction to: J Transl Med (2020) 18:391 10.1186/s12967-020-02509-3

Following publication of the original article [1], the authors identified an error in Fig. 4d. This panel contains cell morphology pictures to show the shape and proliferation capacity of different groups. However, the authors noticed that they’ve mistakenly placed two pictures from the same group into different groups. The correct complete Fig. 4 and its caption are given below and the original article has been corrected.

**Fig. 4:**
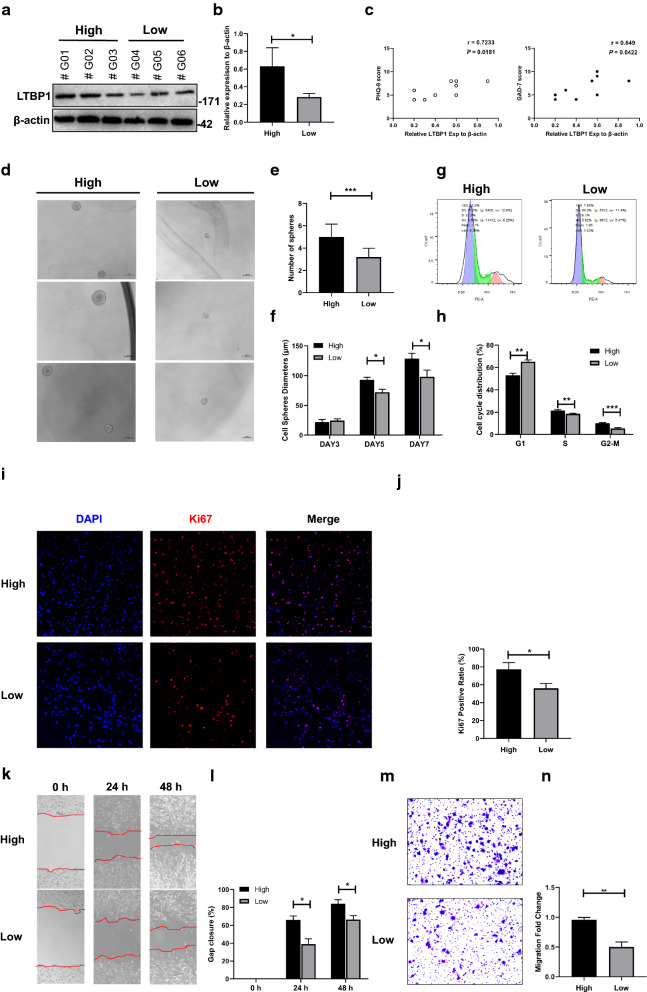
Cellular experiments showed LTBP1 could affect the function of GBM cells. **a–b** Western blotting was used to divide the primary GBM cell into high and low LTBP1 expression groups. β-actin was used as internal reference. *P<0.05; **c** The expression of LTBP1 were positively correlated with PHQ-9 and GAD-7 scores (n = 10). **d** Phase contrast pictures showed the morphology of spheres in both groups. Scale bar represented 200 mm. **e** The number of spheres in each well was counted on days 7, Data were presented as the mean ± SEM; Student’s t-test was chosen as the statistical method; n = 3, *P* = 0.0007 ***P < 0.001. **f** The diameter of spheres was measured to represent the volume. The diameters of spheres in both two groups increased gradually within 3, 5, 7 days, but the diameter of spheres in LTBP1 high expression group grew significantly faster. Data were presented as the mean ± SEM, one-way ANOVA was chosen as the statistical method. n = 3, *P < 0.05. **g–h** Cell cycle of two groups were detected by flow cytometry. A lower percentage of G1 phase, while higher percentage of S and G2-M phase were observed in high LTBP1 expression group than those in low LTBP1 expression group. **P < 0.01. ***P < 0.001. **i–j** Immunofluorescence showed more Ki-67 positive cells in high LTBP1 expression group than low LTBP1 expression group. n = 3, *P* = 0.0168. *, *P *< 0.05. **k–l** Wound healing assay showed faster migration capacity in LTBP1 high expression group than those in LTBP1 low expression group. *P < 0.05. **m–n** Transwell assay showed significantly better migration capacity in high LTBP1 group than those in Low LTBP1 group. n = 3, *P* = 0.0012
