# Supplementary material for: LTBP1 plays a potential bridge between depressive disorder and glioblastoma
Source: J Transl Med. 2020 Oct 15;18:391. doi: 10.1186/s12967-020-02509-3 (PMC7566028; doi:10.1186/s12967-020-02509-3)
Supplement: Supplementary file 1 — Additional file 1: Table S1. Detail items and severity of two surveys. [file 12967_2020_2509_MOESM1_ESM.docx]

**Table S1. Detail items and severity of two surveys**

| Patient Health Questionnaire 9-item (PHQ-9) scale | | Generalized Anxiety Disorder 7-item (GAD-7) scale |
| --- | --- | --- |
| No. | Items of PHQ-9 (n = 9) | Items of GAD-7 (n = 7) |
| 1. | Little interest or pleasure in doing things | Feeling nervous, anxious, or on edge |
| 2. | Feeling down, depressed, or hopeless | Not being able to stop or control worrying |
| 3. | Trouble falling or staying asleep, or sleeping too much | Worrying too much about different things |
| 4. | Feeling tired or having little energy | Trouble relaxing |
| 5. | Poor appetite or overeating | Being so restless that it's hard to sit still |
| 6. | Feeling bad about yourself or that you are a failure or have let yourself or your family down | Becoming easily annoyed or irritable |
| 7. | Trouble concentrating on things, such as reading the newspaper or watching television | Feeling afraid as if something awful might happen |
| 8. | Moving or speaking so slowly that other people could have noticed. Or the opposite being so figety or restless that you have been moving around a lot more than usual |  |
| 9. | Thoughts that you would be better off dead, or of hurting yourself |  |

**Table S1.** The detail of PHQ-9 scale was provided in a 9-item questionnaire. Each item contains four levels (0, 1, 2 and 3). And total PHQ-9 (sum up of all items) refers to the depression severity: **Ⅰ**: 1-4 Minimal depression; **II:** 5-9 Mild depression; **Ⅲ:** 10-14 Moderate depression; **Ⅳ:** 15-19 Moderately severe depression; **Ⅴ:** 20-27 Severe depression. The detail of GAD-7 scale was provided in a 7-item questionnaire. *Each item contains four levels (0, 1, 2 and 3). And total GAD-7 (sum up of all items) refers to the depression severity: **Ⅰ:** 1-4 Minimal Anxiety; **II:** 5-9 Mild Anxiety; **Ⅲ:**10-14 Moderate Anxiety; **Ⅳ:** 15-21 Severe Anxiety.
