# Supplementary material for: LTBP1 plays a potential bridge between depressive disorder and glioblastoma
Source: J Transl Med. 2020 Oct 15;18:391. doi: 10.1186/s12967-020-02509-3 (PMC7566028; doi:10.1186/s12967-020-02509-3)
Supplement: Supplementary file 2 — Additional file 2: Table S2. The severity distribution of PHQ-9 and GAD-7 in enrolled GBM patients. [file 12967_2020_2509_MOESM2_ESM.docx]

**Table S2. The severity distribution of PHQ-9 and GAD-7 in enrolled GBM patients**.

| Survey | Grade | Number of Cases | Score (mean± SE) | Percentage |
| --- | --- | --- | --- | --- |
| PHQ-9 | Ⅰ | 19 | 3.89 ± 0.46 | 26% |
|  | II | 41 | 6.73 ± 1.66 | 56% |
|  | Ⅲ | 13 | 10.92 ± 1.04 | 18% |
|  | Ⅳ | 0 | - | 0 |
|  | Ⅴ | 0 | - | 0 |
| GAD-7 | Ⅰ | 31 | 3.32 ± 0.87 | 42% |
|  | II | 40 | 6.90 ± 1.44 | 55% |
|  | Ⅲ | 2 | 10.00 ± 0.00 | 2.7% |
|  | Ⅳ | 0 | - | 0 |

**Table S2.** * Ⅰ to Ⅴ stands for the different level of the PHQ-9 and GAD-7 questionnaire score, namely, minimal, mild, moderate, etc. Data were presented as Mean ± Standard Deviation (SD); the difference between the two group were analyzed with unpaired sample t test. Abbreviations: GBM: glioblastoma multiforme; CI: confidence interval;
