# Supplementary material for: LTBP1 plays a potential bridge between depressive disorder and glioblastoma
Source: J Transl Med. 2020 Oct 15;18:391. doi: 10.1186/s12967-020-02509-3 (PMC7566028; doi:10.1186/s12967-020-02509-3)
Supplement: Supplementary file 3 — Additional file 3: Figure S1. Overlapped results of PHQ-9 and GAD-7 among GBM patients. a. Pie charts revealed that 36 and 37 patients gained high and low PHQ-9 scores; while 32 and 41 patients gained high and low GAD-7 scores. 19 and 25 patients gained both high and both low PHQ-9 and GAD-7 scores. b. Kaplan Meier plot showed that those patients with both higher PHQ-9 and GAD-7 scores (referred as High-scored) had significantly worse outcome than those with both lower PHQ-9 and GAD-7 scores (P = 0.0005). Figure S2. Bioinformatic mining screened out six potential genes which is involved in both depressive/anxiety disorders and GBM. a Significant difference of outcome could be observed between high and low expression of these six genes. Log-rank (Mantel-Cox) test was used as statistical methods. P ANKK1= 0.016, P FGFR1 = 0.042, P NRG1= 0.0062, P BICC1= 0.039 b Heatmap and volcano plot shows the differentially expressed genes between high and low expression of the six genes. c The expression of these six genes between tumor and normal tissue revealed significantly different expression of LTBP1 in GBM tissue than that in normal tissue. Wilcoxon test was used as statistical methods. P ANKK1= 0.029 (higher expression in normal tissue), P FGFR1 = 0.37, P NRG1= 0.69, P BICC1= 0.29. Figure S3. The hub genes differential expression of which did not influence the outcome of GBM. The hub genes COLA3, LUM, COL2A1, PCOLCE, COL21A1, COL20A1, ASPN and TNMD were not a significant indicator of the outcome in GBM patients. Figure S4 Protein-protein interactions (PPI) related to LTBP1 screened out with only “experimental evidence” selected in STRING database. [file 12967_2020_2509_MOESM3_ESM.pptx]

## Slide 1
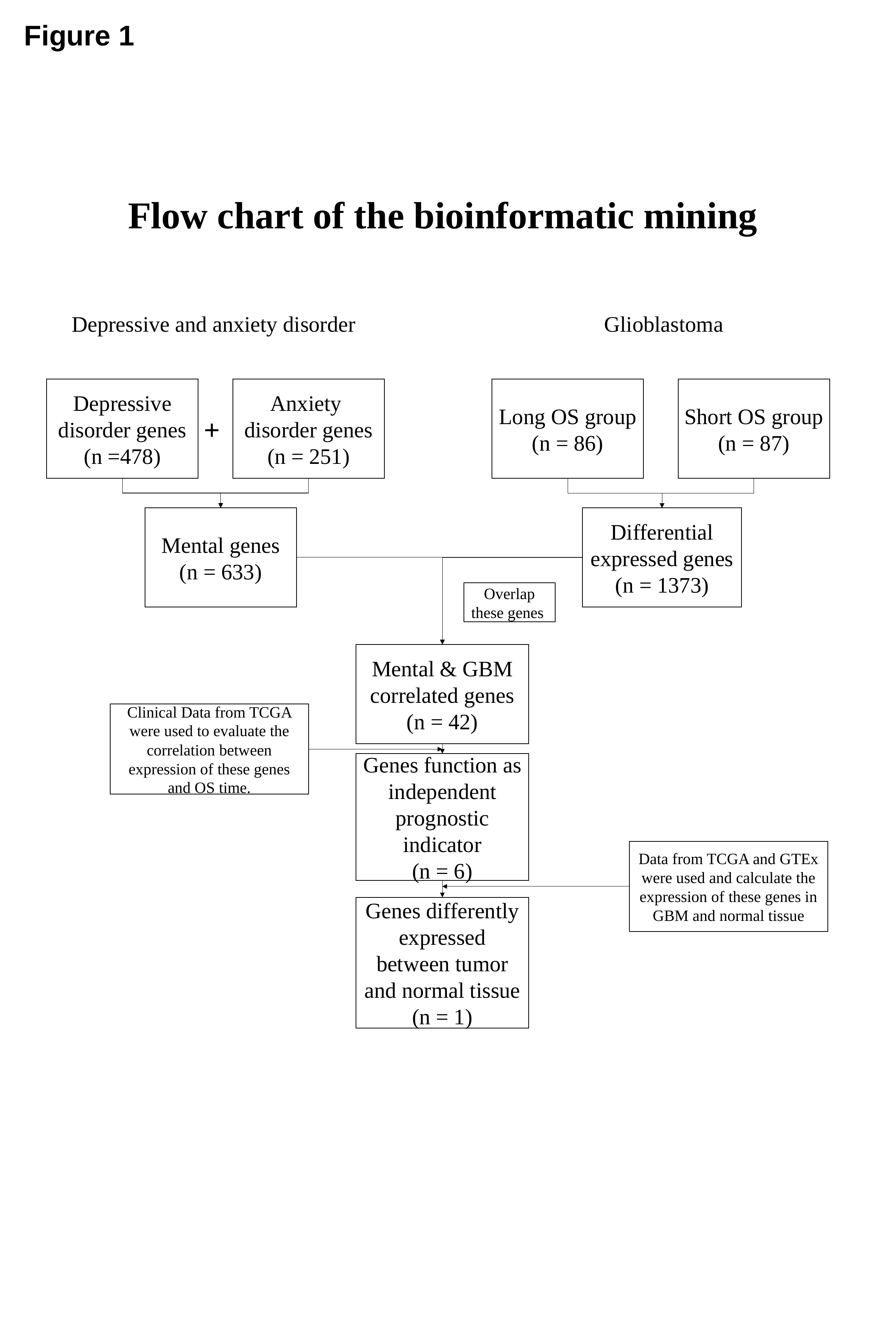

Figure 1
Flow chart of the bioinformatic mining
Depressive and anxiety disorder
Glioblastoma
Depressive disorder genes
(n =478)
Anxiety
disorder genes
(n = 251)
Long OS group
(n = 86)
Short OS group
(n = 87)
+
Mental genes
(n = 633)
Differential expressed genes
(n = 1373)
Overlap these genes
Mental & GBM correlated genes
(n = 42)
Clinical Data from TCGA were used to evaluate the correlation between expression of these genes and OS time.
Genes function as independent prognostic indicator
(n = 6)
Data from TCGA and GTEx were used and calculate the expression of these genes in GBM and normal tissue
Genes differently expressed between tumor and normal tissue (n = 1)

## Slide 2
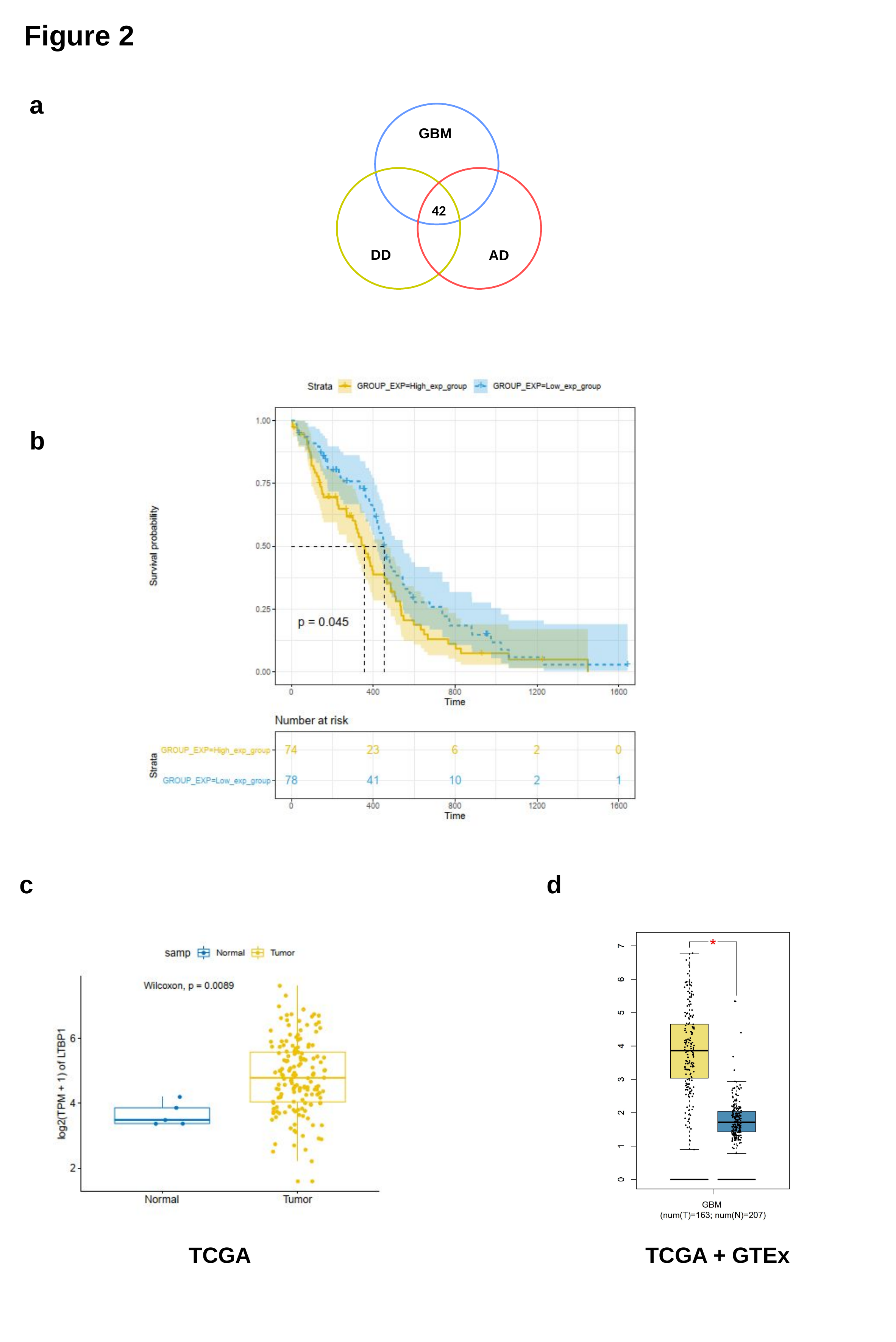

Figure 2
a
GBM
42
DD
AD
b
d
c
TCGA
TCGA + GTEx

## Slide 3
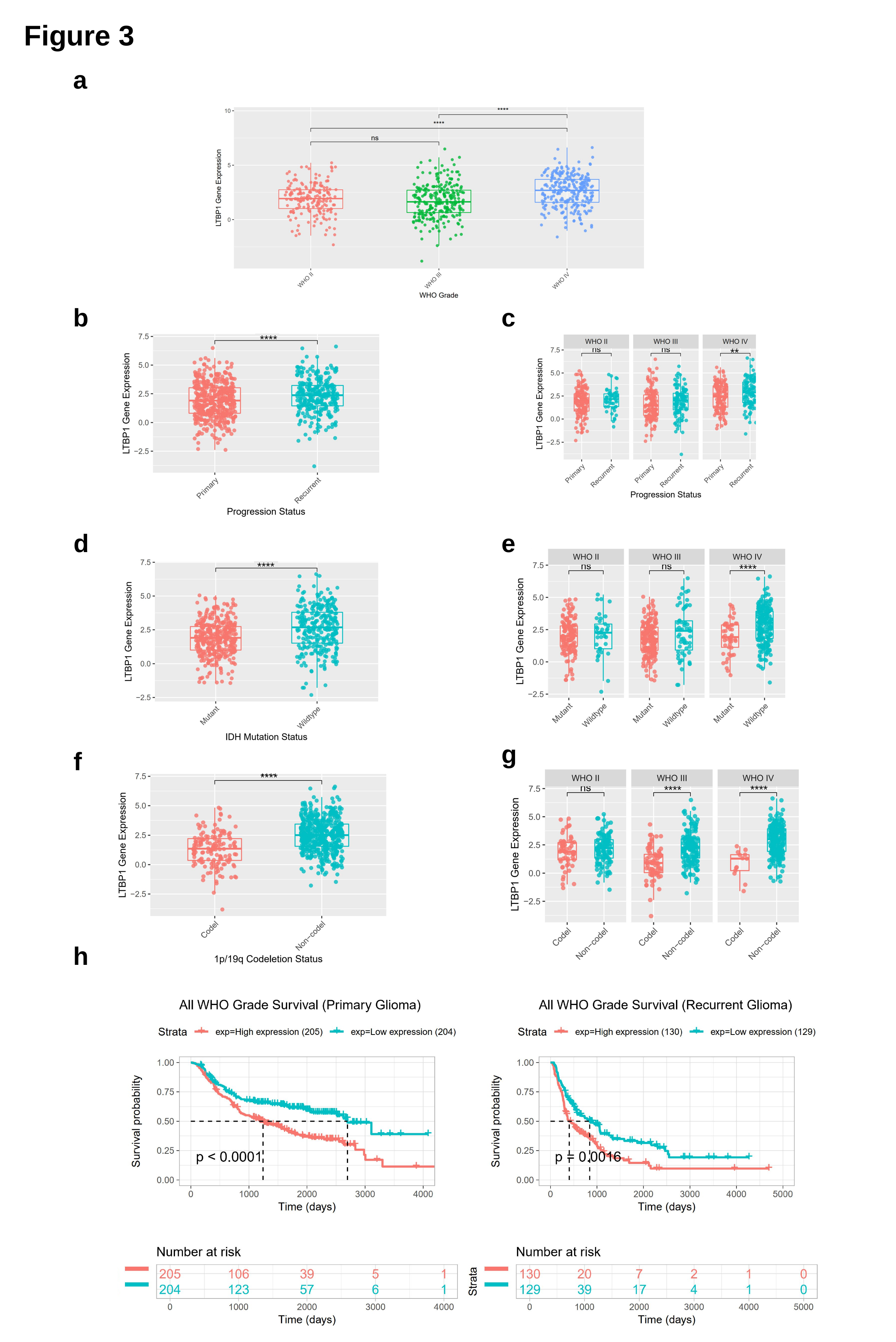

Figure 3
a
b
c
****
**
d
e
****
****
****
g
f
****
****
****
h

## Slide 4
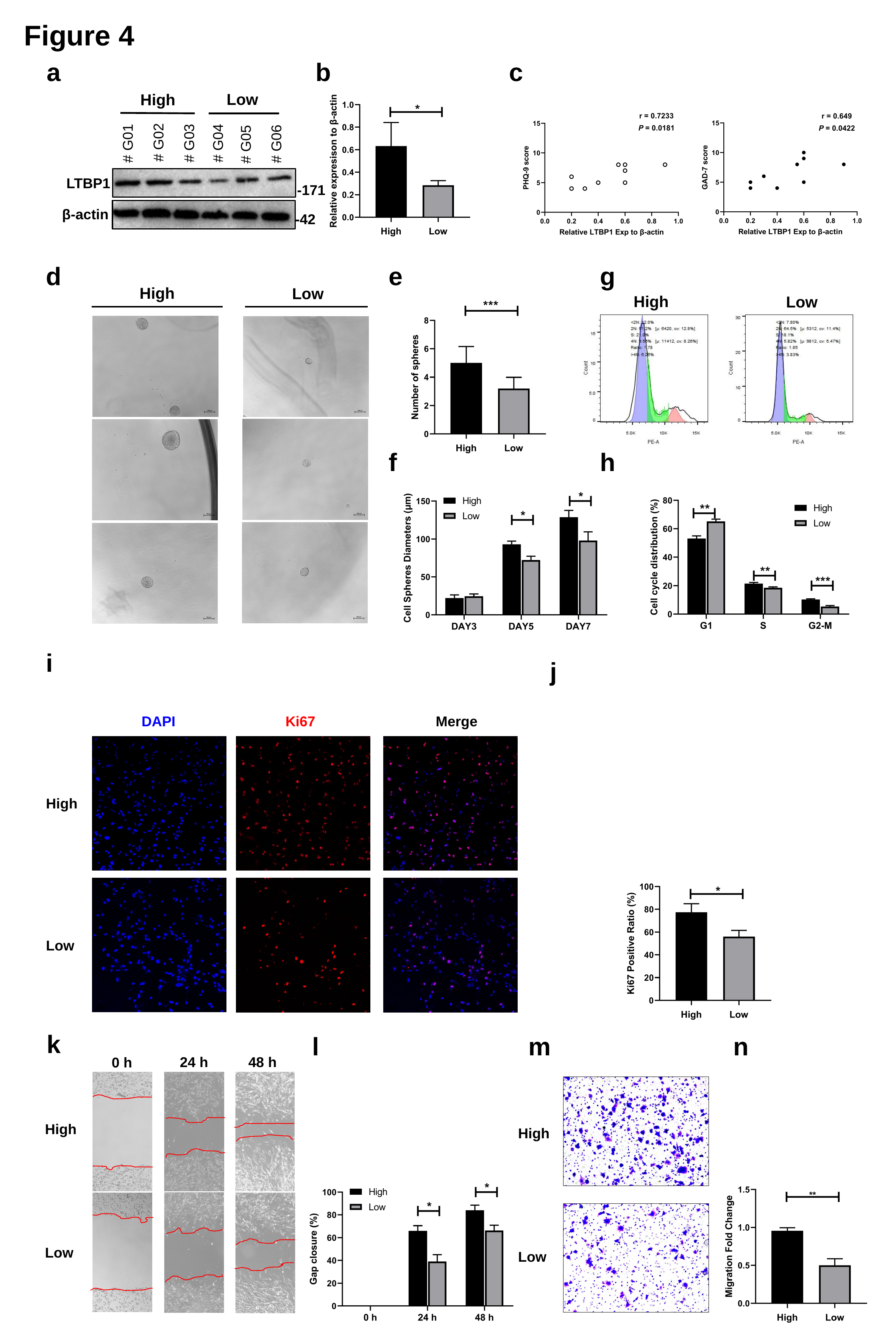

Figure 4
a
b
c
Low
High
# G02
# G03
# G04
# G01
# G06
# G05
β-actin
LTBP1
-171
-42
e
g
d
High
Low
High
Low
f
h
i
j
Ki67
Merge
DAPI
High
Low
k
n
l
m
24 h
48 h
0 h
High
Low
High
Low

## Slide 5
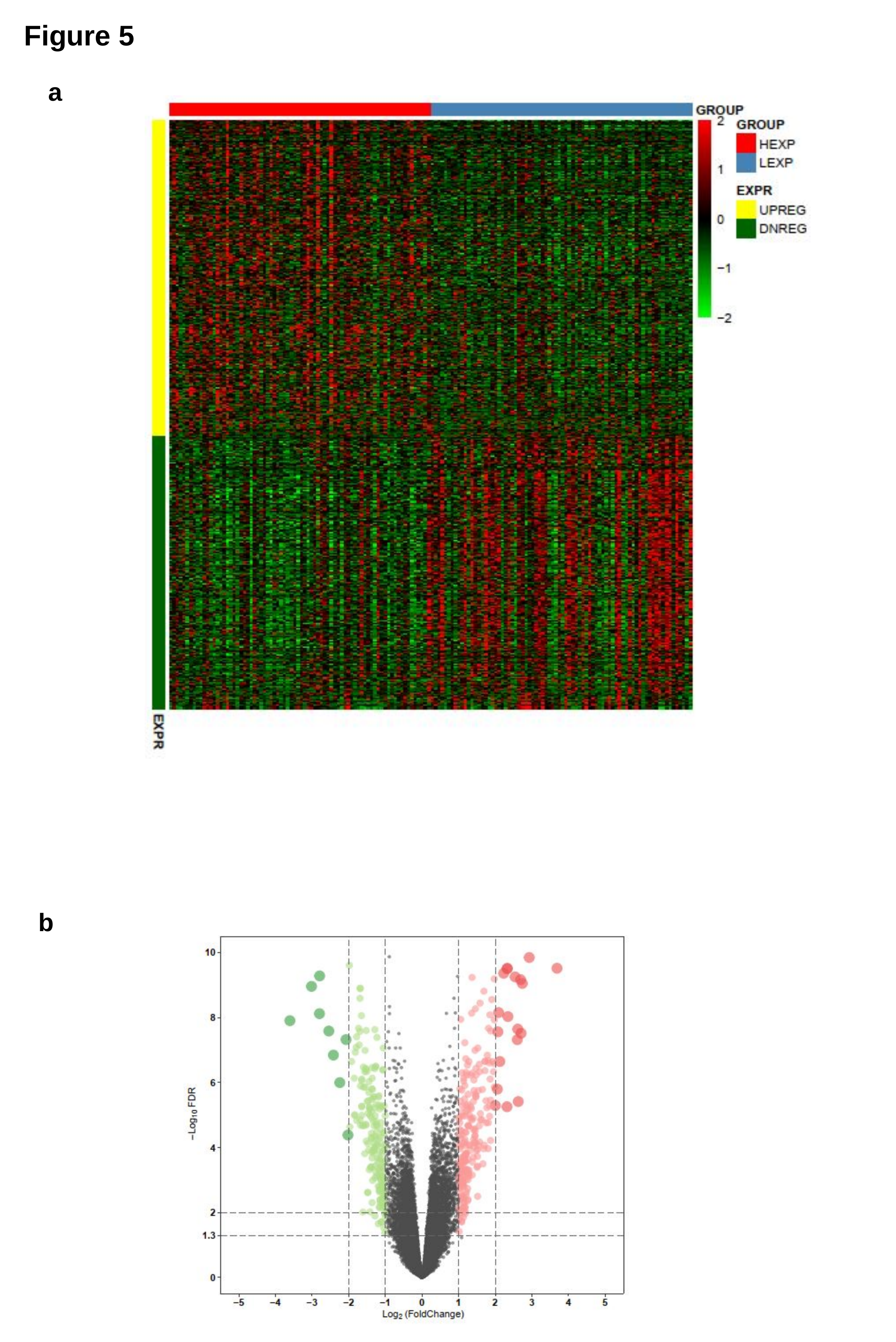

Figure 5
a
b

## Slide 6
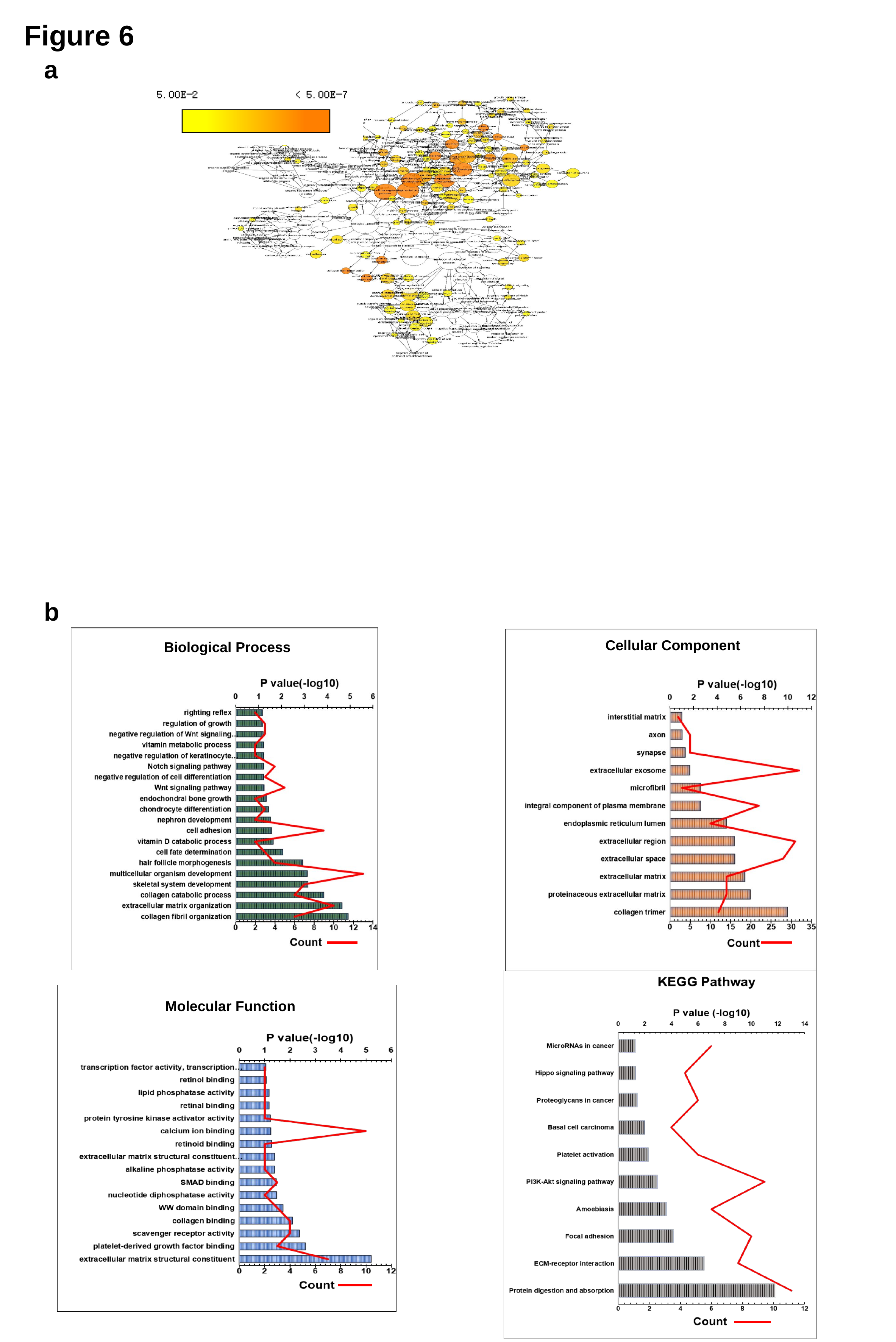

Figure 6
a
b
Biological Process
Cellular Component
Molecular Function

## Slide 7
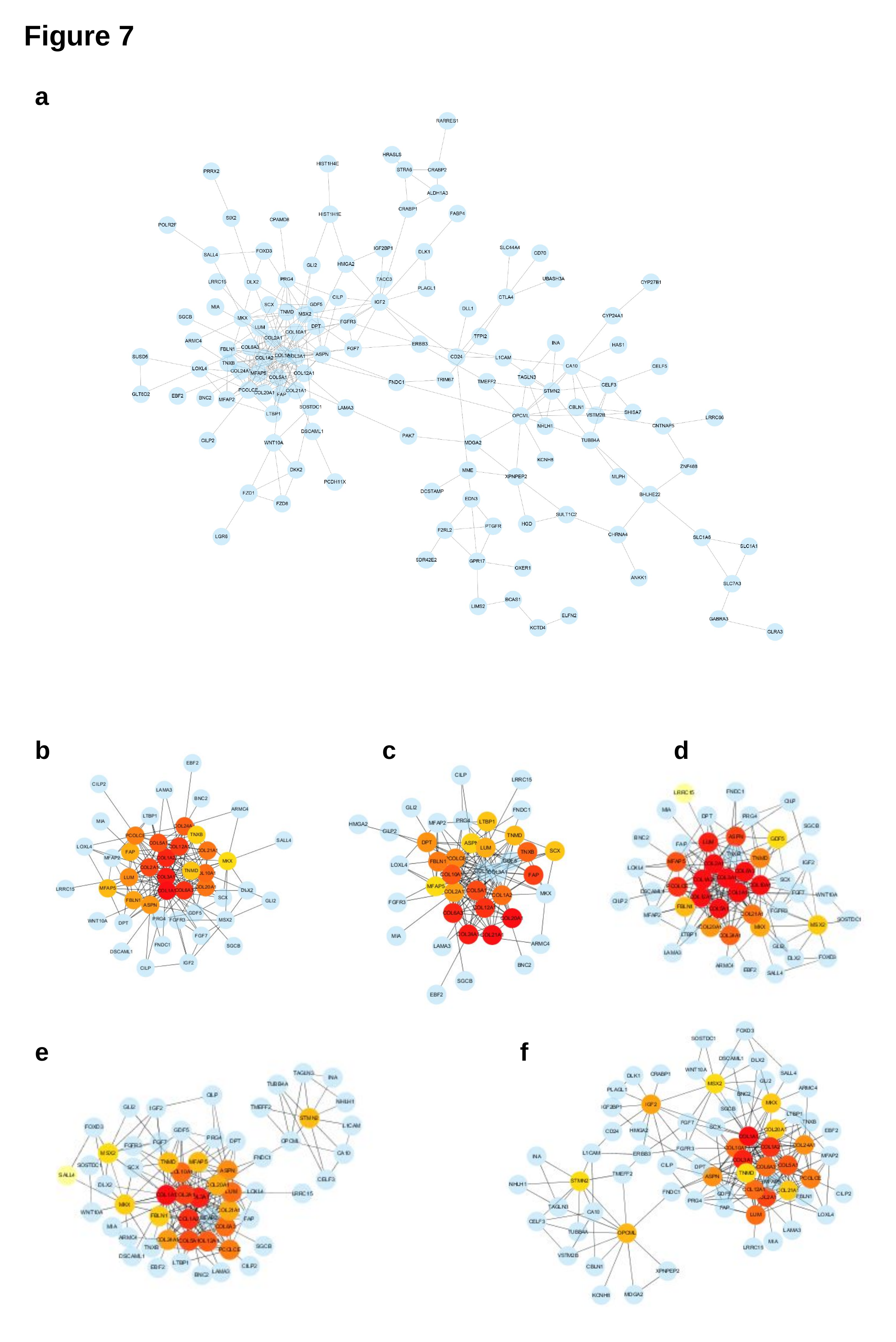

Figure 7
a
b
c
d
e
f

## Slide 8
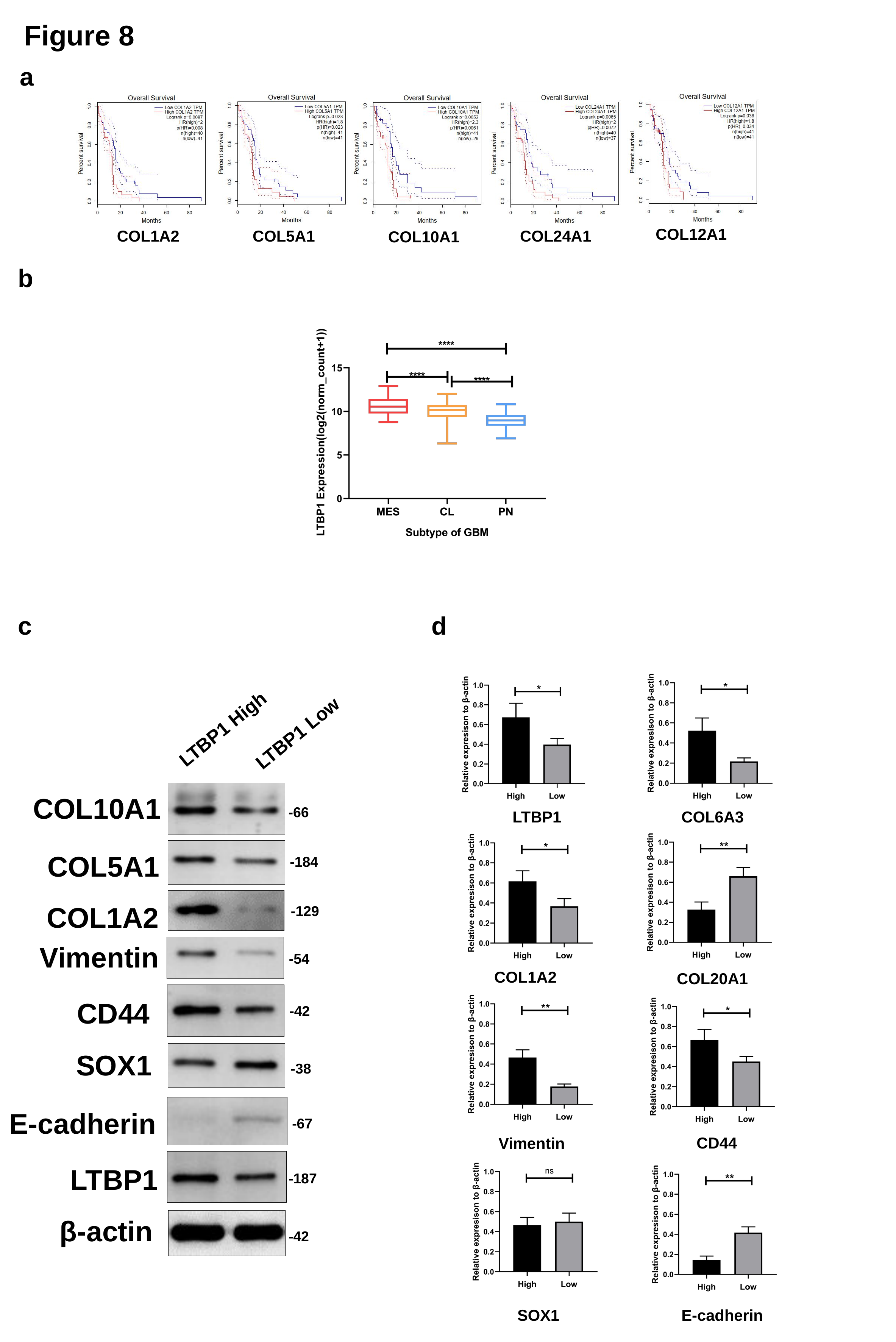

Figure 8
a
COL12A1
COL1A2
COL5A1
COL24A1
COL10A1
b
c
d
COL6A3
COL1A2
COL20A1
CD44
Vimentin
SOX1
E-cadherin
LTBP1
LTBP1 High
LTBP1 Low
COL10A1
COL5A1
COL1A2
Vimentin
CD44
SOX1
LTBP1
β-actin
E-cadherin
-66
-184
-129
-54
-42
-38
-67
-187
-42

## Slide 9
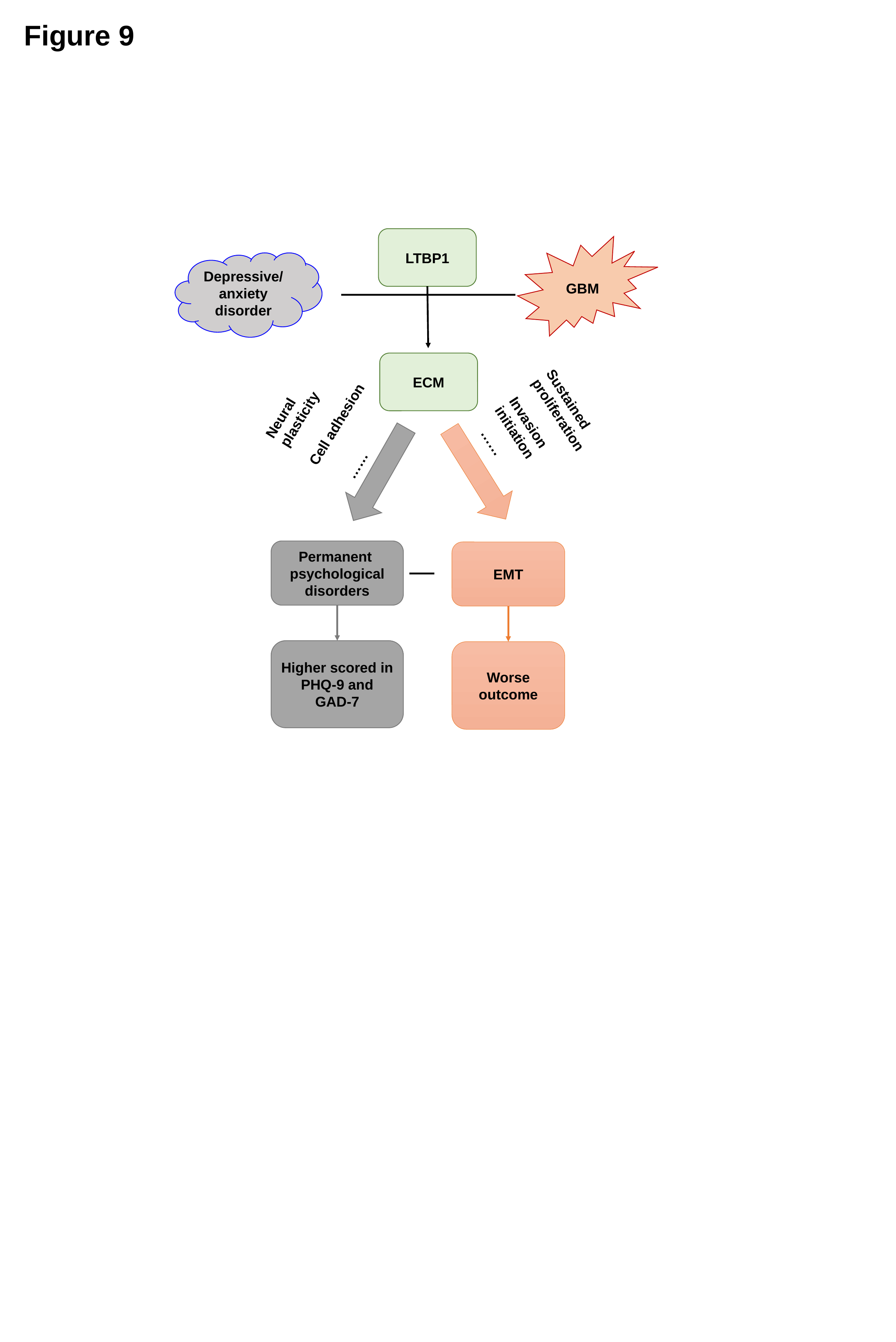

Figure 9
LTBP1
GBM
Depressive/anxiety disorder
ECM
Neural plasticity
Cell adhesion
……
Sustained proliferation
Invasion initiation
……
Permanent psychological disorders
EMT
Higher scored in PHQ-9 and GAD-7
Worse outcome

## Slide 10
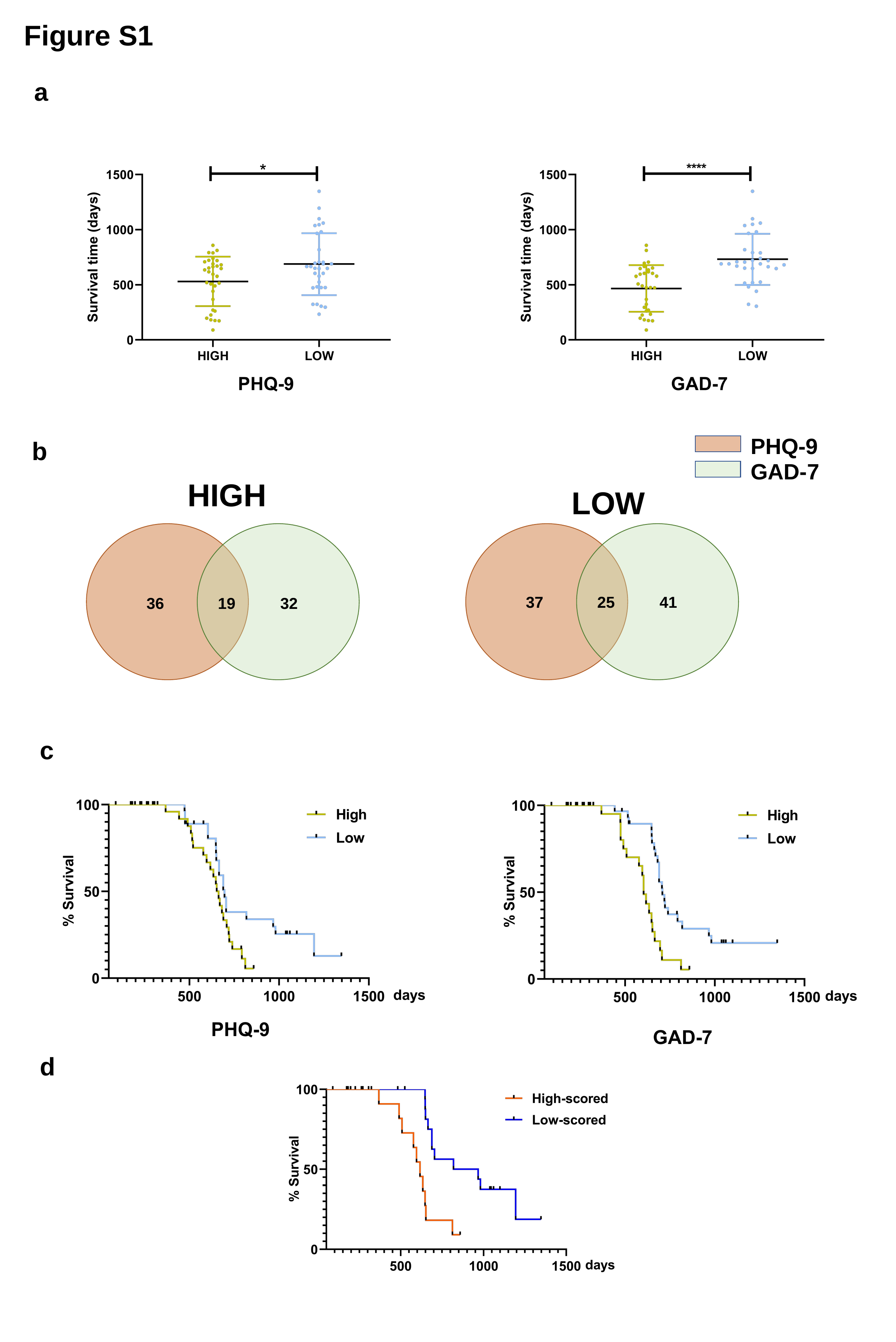

Figure S1
a
PHQ-9
GAD-7
b
HIGH
36
19
32
LOW
37
25
41
c
d

## Slide 11
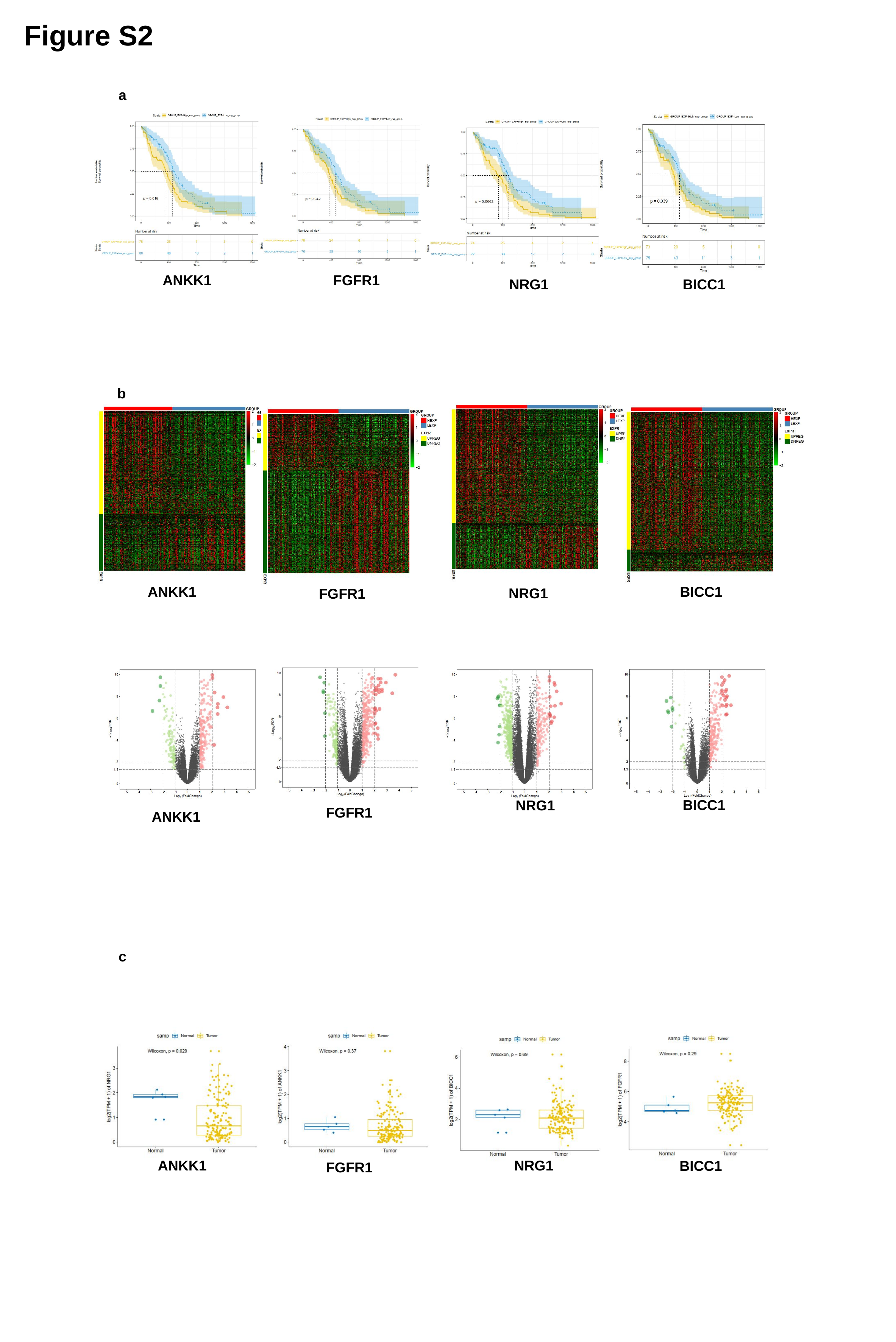

Figure S2
a
BICC1
ANKK1
FGFR1
NRG1
b
D
ANKK1
BICC1
NRG1
FGFR1
BICC1
NRG1
FGFR1
ANKK1
c
ANKK1
NRG1
BICC1
FGFR1

## Slide 12
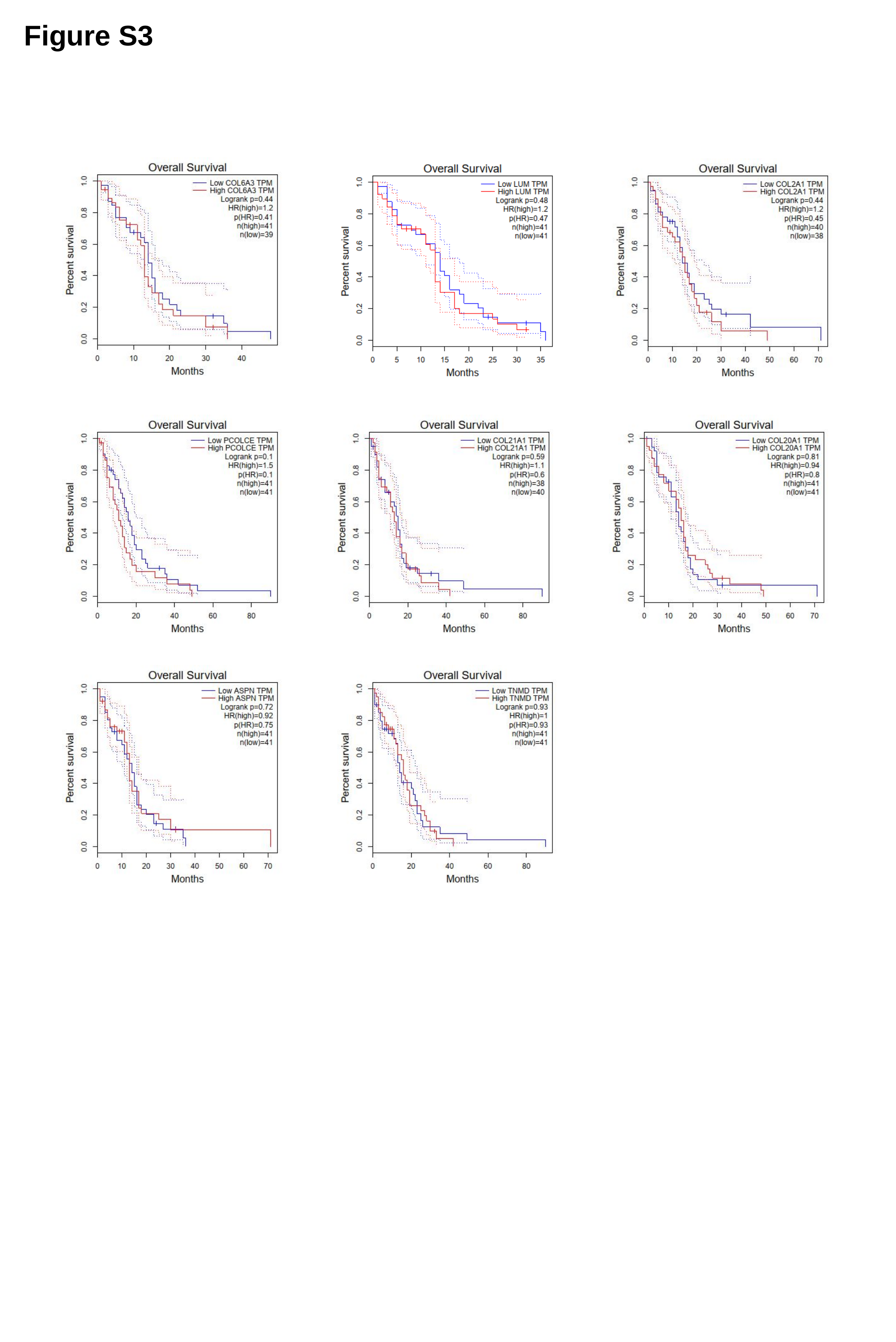

Figure S3

## Slide 13
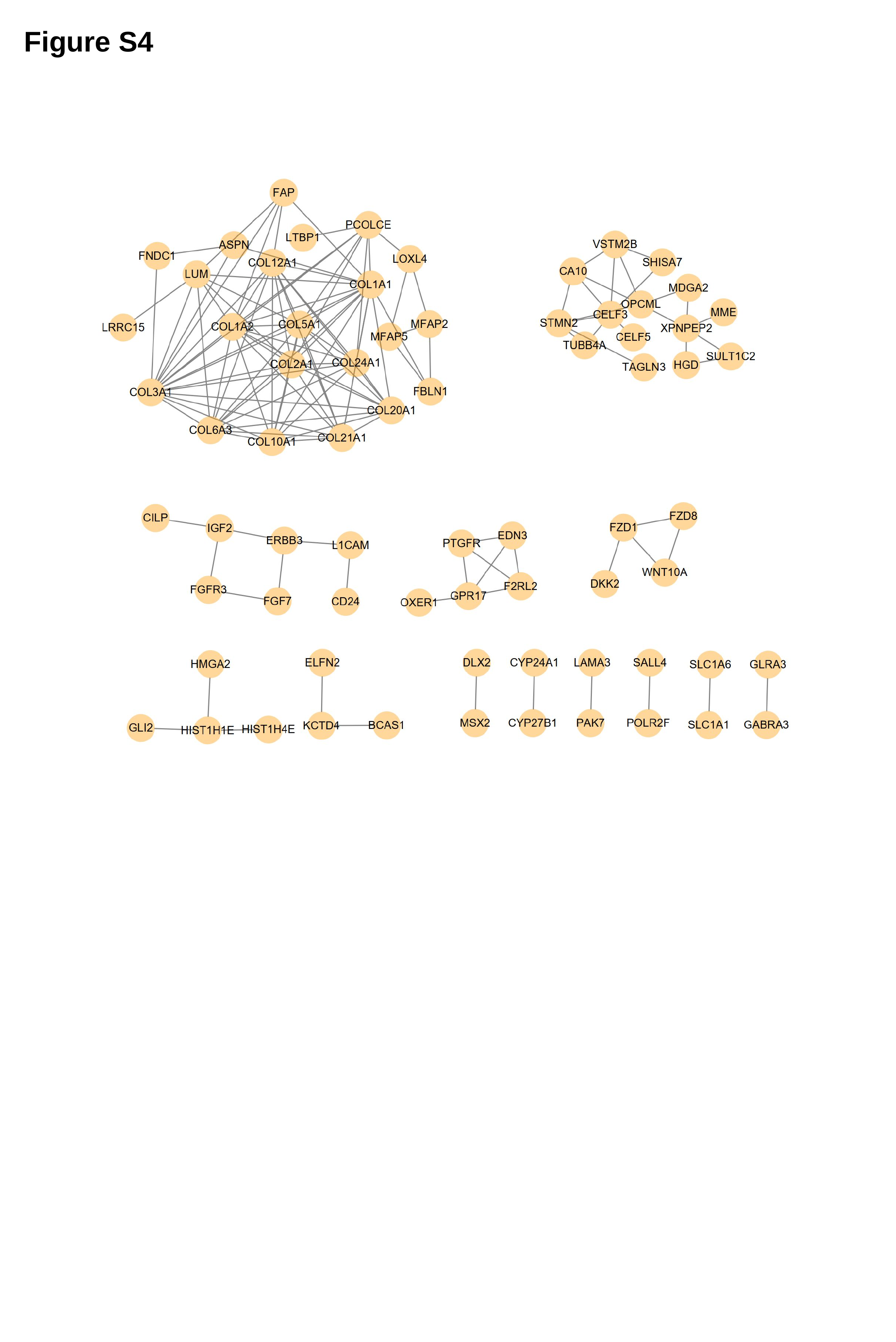

Figure S4
